# Viscoelastic damage evaluation of the axon

**DOI:** 10.3389/fbioe.2022.904818

**Published:** 2022-10-06

**Authors:** Fuad Hasan, KAH Al Mahmud, Md. Ishak Khan, Ashfaq Adnan

**Affiliations:** ^1^ Department of Mechanical and Aerospace Engineering, The University of Texas at Arlington, Arlington, TX, United States; ^2^ Perelman School of Medicine, University of Pennsylvania, Philadelphia, PA, United States

**Keywords:** mechanical behavior of axon, neuron, cytoskeleton, traumatic brain injury, finite element analysis, representative volume element (RVE), composite materials, mechanical characterization

## Abstract

In this manuscript, we have studied the microstructure of the axonal cytoskeleton and adopted a bottom-up approach to evaluate the mechanical responses of axons. The cytoskeleton of the axon includes the microtubules (MT), Tau proteins (Tau), neurofilaments (NF), and microfilaments (MF). Although most of the rigidity of the axons is due to the MT, the viscoelastic response of axons comes from the Tau. Early studies have shown that NF and MF do not provide significant elasticity to the overall response of axons. Therefore, the most critical aspect of the mechanical response of axons is the microstructural topology of how MT and Tau are connected and construct the cross-linked network. Using a scanning electron microscope (SEM), the cross-sectional view of the axons revealed that the MTs are organized in a hexagonal array and cross-linked by Tau. Therefore, we have developed a hexagonal Representative Volume Element (RVE) of the axonal microstructure with MT and Tau as fibers. The matrix of the RVE is modeled by considering a combined effect of NF and MF. A parametric study is done by varying fiber geometric and mechanical properties. The Young’s modulus and spacing of MT are varied between 1.5 and 1.9 GPa and 20–38 nm, respectively. Tau is modeled as a 3-parameter General Maxwell viscoelastic material. The failure strains for MT and Tau are taken to be 50 and 40%, respectively. A total of 4 RVEs are prepared for finite element analysis, and six loading cases are inspected to quantify the three-dimensional (3D) viscoelastic relaxation response. The volume-averaged stress and strain are then used to fit the relaxation Prony series. Next, we imposed varying strain rates (between 10/sec to 50/sec) on the RVE and analyzed the axonal failure process. We have observed that the 40% failure strain of Tau is achieved in all strain rates before the MT reaches its failure strain of 50%. The corresponding axonal failure strain and stress vary between 6 and 11% and 5–19.8 MPa, respectively. This study can be used to model macroscale axonal aggregate typical of the white matter region of the brain tissue.

## Introduction

Under dynamic loading (e.g., rotational acceleration), the human brain tissue experiences shear deformation in different length scales (Wright and Ramesh, 2012), (Hasan et al., 2021a). The tissue level deviatoric deformation leads to cellular level stretching. The degree of stretching depends on the orientation and location of the individual axons. The rotational acceleration plane, the interface between white matter and gray matter, and the presence of stiff membranes can cause significant stress concentration at the cellular level (Smith and Meaney, 2000). This type of loading condition may lead to mechanical failure of the axonal microstructure and cause the most common pathological feature of Traumatic Brain Injury (TBI) called Diffuse Axonal Injury (DAI) (al Mahmud et al., 2020)– (Hasan et al., 2021b). However, we cannot directly measure or investigate DAI with the current technology (Wright, 2012). It is crucial to understand how the mechanical load is transferred from macro to microscale and to identify injury thresholds based on the cytoskeletal component failure. A multiscale computational approach is needed to bridge the injury information to measurable means. To understand how continuum scale tissue damage is correlated to the cellular microstructure, in this manuscript, we have studied and characterized the mechanical behavior of the axonal cytoskeletal core.

**FIGURE 1:**
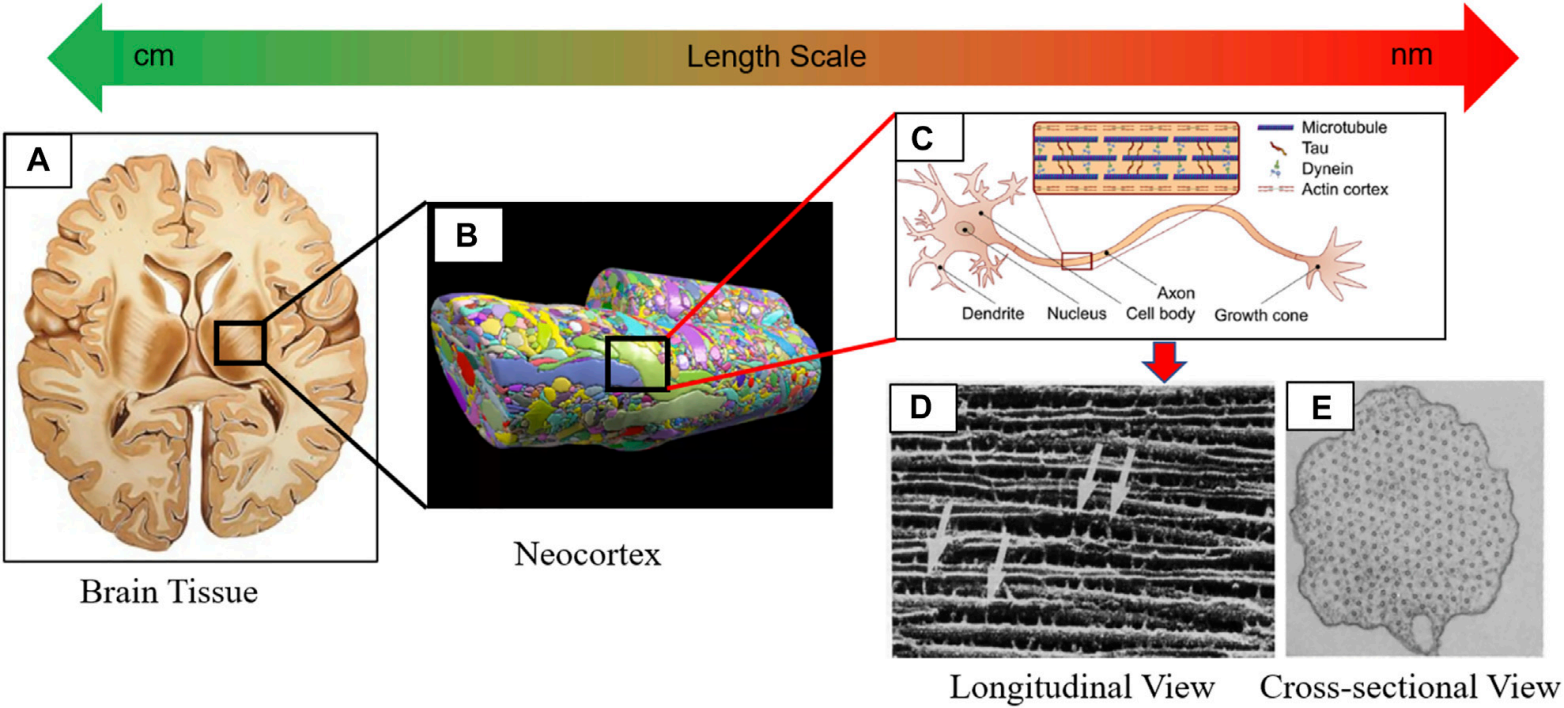
Depicting multiscale hierarchical structure of the brain tissue ranging from macroscale tissue to microscale neuronal cells. **(A)** Cross-sectional view of the brain tissue (gray and white matter) in the horizontal plane. **(B)** Reconstruction of the neocortex shows the heterogeneity posed by neuronal cells and ECM (Kasthuri et al., 2015). **(C)** Anatomy of the neuronal cell (de Rooij et al., 2017). **(D)** SEM image shows how axially oriented MT are cross-linked with the Tau. **(C)** A cross-sectional view of the transverse plane shows the hexagonal orientation of MT. **(C–E)** are adapted from (Chen et al., 1992)).

The brain is primarily classified into two regions at the tissue level: 1) gray matter and 2) white matter (see Figure 1A). The neuronal cells are randomly distributed in the gray matter giving its isotropic mechanical response (see Figure 1B); (Koser et al., 2015). The short-term shear modulus of the gray matter varies from (Kleiven, 2007)- (Shamloo et al., 2015) varies from 10–34 kPa (Zhang et al., 2004)– (Taylor and Ford, 2009). Neuronal cells, bundled together to form fiber tracts, are more organized in the white matter region. Therefore, the mechanical behavior is anisotropic in the white matter (Eskandari et al., 2021). The anisotropy can be introduced to the materials modeling of the white matter using diffusion tensor imaging (DTI) (Wright, 2012). One typical feature of white matter is that the glial cells extend their lipid bilayer and wrap the neurons called the myelin sheaths. Due to this intercellular network of the glial cells, white matter shows higher stiffness than gray matter (Weickenmeier et al., 2016). Coarse grain molecular dynamic simulations were performed to estimate the bulk modulus and Poisson’s ratio of lipid bilayer in the range of 21–54 MPa and 0.11–0.44, respectively. (el Sayed et al., 2008).-0.44, respectively (Jadidi et al., 2014), (Ayton et al., 2002). The excessive presence of the lipid bilayer in the myelin sheath could be another reason for higher stiffness in the white matter. The typical short-term shear modulus of the white matter region varies between (WATANABE et al., 2009)- (Chen et al., 1992) varies between 12–42 kPa (Zhang et al., 2004)– (Taylor and Ford, 2009). Several studies focused on characterizing white matter tissue properties in a bottom-up multiscale approach. In those studies, axon, and extracellular matrix (ECM) properties were used as input parameters, and a micromechanical method was used to estimate the effective properties (Arbogast and Margulies, 1999), (Yousefsani et al., 2018). In another study, Samad et al. (2013) used a top-down approach. The effective properties of axon and ECM were estimated using the white matter tissue-level properties from the relaxation tests (Javid et al., 2014). They have reported the short-term shear modulus of axon and ECM as 12.86 and 4.29 kPa, respectively. Similar stiffness of axon was also reported by Robert et al. (2007), who used the microneedle technique to stretch the PC12 neurites and found the elastic modulus to be 12 kPa (Bernal et al., 2007).

The inherent mechanical stiffness of the axon is due to its cytoskeletal components consisting of microtubule (MT), microfilament (MF), and neurofilament (NF) (see Figure 1C). Notably, a hexagonal array of MTs constructs a cross-linked network of viscoelastic nature with microtubule-associated protein tau (Tau) (see Figures 1D,E). Further study on the contribution of the axonal cytoskeleton components to the elastic properties of the axon was performed by Ouyang et al. (2013) (Ouyang et al., 2013). They carefully disrupted the individual cytoskeletal members by using Nocodazole, Cytochalasin D, and Acrylamide to disrupt MT, MF, and NF, respectively. When no drug was used, the transverse elastic modulus of the axon was found to be 9.5 kPa. The axonal elastic modulus was calculated from the atomic force microscopy (AFM) as 1.4, 5.7, and 3.4 kPa, respectively, by separately disrupting the MT, MF, and NF. It was concluded that the maximum stiffness of the axon came from the contribution of the MT cross-linked network. However, when all drugs were used, the elastic modulus of the axon was estimated to be 2 kPa which was very close to the computational estimation of the axonal membrane stiffness of 4.23 kPa (Zhang et al., 2017). Several studies were directed toward studying mechanical responses of axonal cytoskeleton components and reviewed in ref. (Khan et al., 2020), (Khan et al., 2021a). The viscoelastic response of MT, MF, NF, and Tau was studied in ref. (Adnan et al., 2018).– (Ishak Khan et al., 2021). Special attention was given to the MT-Tau network response in various loading conditions (e.g., tension, torsion) (Tang‐Schomer et al., 2010)– (Wu et al., 2019). Besides the structural failure, a cascade of complex molecular events follows the mechanical insult. The effect of phosphorylation initiates MT-Tau network disintegration much earlier than the fibers (e.g., MT and Tau) reach their failure strain (Khan et al., 2021c).

As discussed here, we can see that the study on the brain tissue mechanics using the top-down multiscale approach ends at the tissue to cellular level bridging. The high-fidelity full head models used the most refined mesh of 1–5.2 mm in size to study TBI using the finite element method (FEM) (Taylor and Ford, 2009), (Panzer et al., 2013)– (Chafi et al., 2010). Even in the lowest volume fraction, there could be thousands of axons within 1 mm mesh which must be resolved. Moreover, experimental observations indicated that the local cellular level strain was about 25% of the tissue level strain (Tamura et al., 2007). The heterogeneity and the interplay of the cellular microstructure is thought to be the reason of the inter-level mismatch (Montanino and Kleiven, 2018). Therefore, the threshold strain at the tissue level related to the TBI cannot be directly used to predict the DAI.

On the other hand, numerous studies were performed at the sub-cellular level. However, there are gaps between the sub-cellular level to the cellular level, and no attempts were made to use the bottom-up approach to connect these two levels. There is a need for bridging the cytoskeletal component responses to the effective properties of neuronal axons. The bridging will allow us to model the intermediate level between the cellular and tissue level, the axon aggregates. Understanding how cellular level strain is transmitted to the cytoskeletal sub-cellular level is also critical. Therefore, in this manuscript, we have addressed these two issues.

In the first part of this manuscript, we performed three-dimensional (3D) micromechanical viscoelastic characterization of the axon cytoskeletal core. We have developed the representative volume element (RVE) of the axonal microstructure for the micromechanical study. As discussed earlier, the primary fibers that carry the mechanical load and give stiffness to the axons are MTs. The MTs are packed unidirectionally in the hexagonal orientation and cross-linked with Tau (Chen et al., 1992). The axoplasm surrounding the MT-Tau cross-linked network is modeled as the matrix of the RVE. We have prepared four RVEs to perform the parametric study on the geometric variables (e.g., Tau radius, Tau spacing, and MT spacing). We have also varied the stiffness of the MT, and comparisons are discussed. Since there are three mutually orthogonal planes of symmetry in the RVE, the 3D mechanical response is orthotropic, and a total of nine viscoelastic relaxation functions are required to fully characterize the axonal core (Naik et al., 2008). As we have planned to use the relaxation functions as input parameters to develop the higher-level axon aggregate model, it is suitable for us to express the relaxation function as the Prony series. Six loading cases have been implemented on the RVE for the nine relaxation functions. Three of them are normal, and the rest are shear loading conditions (Wang et al., 2017). The homogenization is done by calculating the volume-averaged stress and strain of the RVE using the FEM (Sun and Vaidya, 1996a); (Barbero). The nonlinear regression fit is done between the volume-averaged stress and the hereditary integral expression of the Prony series coefficients by using the Levenberg-Marquardt algorithm (Gavin, 2019a), (Tzikang and Chen, 2000).

In the second part of this manuscript, we evaluated the axonal cytoskeletal damage threshold by varying the loading rates. The criteria for damage threshold are set to the MT and Tau failure strain of 50 and 40%, respectively (Janmey et al., 1991)– (Wegmann et al., 2011). Based on the previous studies, we have varied the strain rate from 10/s to 50/s with a 10/s increment in the direction of MT orientation (Elkin and Morrison, 2007)– (Morrison et al., 2003). The corresponding volume-averaged failure stress and strain of the axonal cytoskeletal core have been calculated and summarized.

## Material and geometric properties of axon

We have taken a bottom-up approach from the multiscale point of view to develop the axon’s representative volume element (RVE) and characterize the mechanical properties. In doing so, we have modeled the detailed cytoskeletal components of the axon. The long stem-like part of the neuronal cell is the axon, and the main mechanical property is due to its microstructural components. Microtubules (MT) are the primary cytoskeletal component from which significant rigidity is contributed (Ouyang et al., 2013). Axially oriented MTs are cross-linked by the tau protein (Tau) of viscoelastic nature. The time-dependent viscoelastic nature of the axon is generally coming from the Tau. Figures 1C–E shows the detail of the microstructural components and their orientation.

**FIGURE 2:**
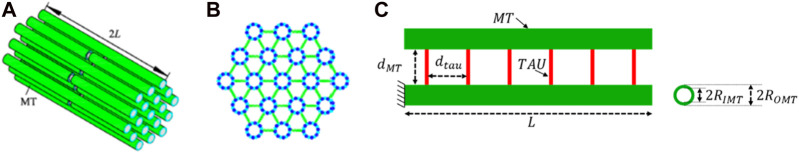
**(A,B)** Axonal microstructural model for analysis (adapted from (Wu et al., 2019)). **(C)** Geometric properties of the MT-Tau cross-linked network.

The cross-sectional view of the axon indicates that the MTs are oriented in a hexagonal array, and most axonal microstructure studies model the MT bundle as such. Figures 2A,B shows how the MT bundles are cross-linked with Tau.

Just like any other microstructure of biomaterials, axonal microstructural parameters vary within a wide range. Figure 2C shows the geometric definition we have used as parameters. Table 1 summarizes those parameters and refers to the literature. The length of the MTs ( 2L ) is between 1 and 10 μm with discontinuity within 80% of the center of the length. The inner ( $R_{IMT}$ ) and the outer ( $R_{OMT}$ ) radii of the MT are 7 and 12.5 nm, respectively. The Young’s modulus of MT ( $E_{MT}$ ) varies between 1.5 and 1.9 GPa. The spacing between two MTs ( $d_{MT}$ ) are within 20–38 nm. MT fails at 50% strain ( $\epsilon_{MT}$ ).

**TABLE 1:** Material and geometric properties of the cytoskeletal components.

Parameters	Quantity	Value	References
$R_{OMT}$	MT Outer Radius	12.5 nm	Pampaloni et al. (2006)
$R_{IMT}$	MT Inner Radius	7 nm	Pampaloni et al. (2006)
$E_{MT}$	MT Young’s Modulus	1.5–1.9 GPa	Soheilypour et al. (2015)
$d_{MT}$	End-to-End MT Spacing	20–38 nm	Rosenberg et al. (2008)
$R_{tau}$	Tau Radius	4–10 nm	Spillantini and Goedert, (1998)
$E_{tau}$	Tau Young’s Modulus (for KV model)	5 MPa	(Soheilypour et al., 2015), (Peter and Mofrad, 2012b)
$\tau_{tau}$	Tau Retardation Time (for KV model)	0.35 s	(Ahmadzadeh et al., 2014), (Wegmann et al., 2011)
$d_{tau}$	Tau-Tau Spacing	20–40 nm	Hirokawa et al. (1988)
2L	MT Length	1–10 μm	(Pampaloni et al., 2006), (Yu and Baas, 1994)
$\epsilon_{MT}$	MT Failure Strain	50%	(Janmey et al., 1991)– (Sarangapani et al., 2013)
$\epsilon_{tau}$	Tau Failure Strain	40%	Wegmann et al. (2011)
$G_{matrix}$	Matrix Shear Modulus	552.63 Pa	Ouyang et al. (2013)

The Tau protein, on the other hand, shows viscoelastic mechanical behavior and is usually modeled as the Kelvin-Voigt model. The radius of Tau $\left(R_{tau}\right)$ varied between 4 and 10 nm. The Young’s modulus of Tau ( $E_{tau}$ ) is 5 MPa and the retardation time ( $\tau_{tau}=\mu_{tau}/E_{tau}$ ) is 0.35 s. Therefore, the Tau viscosity is measured to be $\mu_{tau}=1.75\;MPa.s$ . The spacing of Tau ( $d_{tau}$ ) is within 20–40 nm. Tau failure strain ( $\epsilon_{tau}$ ) is 40%.

**FIGURE 3:**
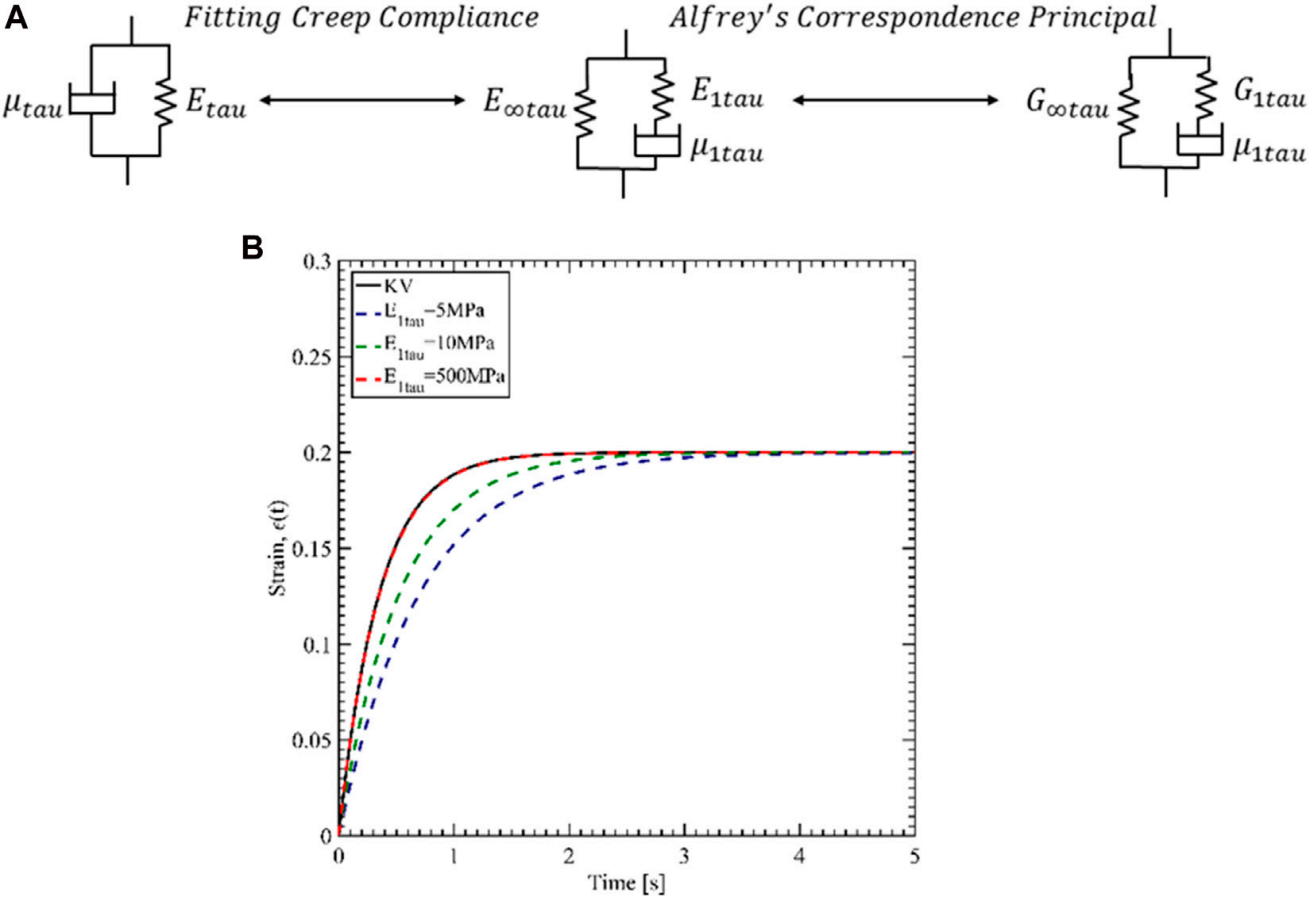
**(A)** Procedure of defining the Tau properties for ANSYS. **(B)** Creep response fit of 3-parameter General Maxwell to Kelvin-Voigt.

As mentioned, the available material data for Tau protein is for the Kelvin-Voigt (KV) model. However, we have used ANSYS Mechanical for the viscoelastic characterization, and ANSYS only accepts viscoelastic parameters for General Maxwell (GM) model (Citation: “Ansys® Academic Research Mechanical, Release 18.1, Help System, Ansys APDL Theory Guide, ANSYS, Inc.”). Another challenge is that the relaxation modulus must be input for shear and bulk modulus. Therefore, we have first estimated the GM parameters for Young’s modulus by fitting creep responses of KV and GM. Then Alfrey’s correspondence principle was utilized to evaluate the shear relaxation parameters for Tau. Figure 3A shows the two steps of finding the parameters.

The constitutive relation between the stress ( σ ) and strain ( ϵ ) for KV and 3-parameter GM model is given below,

$$KV:\sigma =E_{tau}\epsilon +\mu_{tau}\dot{\epsilon }$$ (1)

$$GM:\sigma +\frac{\mu_{1tau}}{E_{1tau}}\dot{\sigma }=E_{\infty tau}\epsilon +\frac{\mu_{1tau}\left(E_{\infty tau}+E_{1tau}\right)}{E_{1tau}}\dot{\epsilon }$$ (2)

Eqs 1, 2 are solved for creep loading ( σ≠0 and $\dot{\sigma }=0$ ) by setting $E_{tau}=E_{\infty tau}$ and $\mu_{tau}=\mu_{1tau}$ . Figure 3B shows the fit by increasing $E_{1tau}$ . From the analysis, the best fit is considered for $E_{1tau}=100E_{tau}$ . The viscoelastic Young’s modulus relaxation function for the 3-parameter GM is then,

$${E\left(t\right)}_{tau}=E_{\infty tau}+E_{1tau}\exp\left(-\frac{t}{\tau_{1tau}}\right)$$ (3)

Where t is the time and the relaxation time for the Maxwell element is $\tau_{1tau}=\mu_{1tau}/E_{1tau}=3.5\;ms$ .

Once we have estimated the values of the 3-parameter GM model for Young’s modulus, we determined the shear modulus values. Alfrey’s correspondence principle states that the viscoelastic modulus is related to the Hookean linear elastic material in the frequency domain (Brinson and Brinson, 2008). In the linear elastic theory, the shear modulus and Young’s modulus is related by,

$$G=\frac{E}{2\left(1+\nu \right)}$$ (4)

In the above equation, G, E, and ν are shear modulus, Young’s modulus, and Poisson’s ratio in the linear elastic domain, respectively. In the frequency domain, Eq. 4 is,

$${\bar{G}}^{*}\left(s\right)=\frac{{\bar{E}}^{*}\left(s\right)}{2\left(1+{\bar{\nu }}^{*}\left(s\right)\right)}$$ (5)

Where, s=a+ib , is a complex function. In Eq. 5, the frequency domain representation of the shear, Young’s, and Poisson’s ratio is related to the time domain representation via the Laplace transformation.

$${\bar{E}}^{*}\left(s\right)=s\int_{0}^{\infty }e^{-st}{E\left(t\right)}_{tau}dt$$

$${\bar{G}}^{*}\left(s\right)=s\int_{0}^{\infty }e^{-st}{G\left(t\right)}_{tau}dt$$ (6)

$${\bar{\nu }}^{*}\left(s\right)=s\int_{0}^{\infty }e^{-st}{\nu \left(t\right)}_{tau}dt$$

Considering constant Poisson’s ratio for Tau, ${\nu \left(t\right)}_{tau}=0.33$ and using Eq. 3, we can substitute Eq. 6 into Eq. 5 to solve the shear relaxation function. The expression for the shear relaxation modulus for Tau is as given below,

$${G\left(t\right)}_{tau}=G_{\infty tau}+G_{1tau}\exp\left(-\frac{t}{\tau_{1tau}}\right)$$ (7)

Where long term shear modulus, ${G\left(\infty \right)}_{tau}=G_{\infty tau}=1.87\;MPa$ , $G_{1tau}=187.97\;MPa$ and $\tau_{1tau}=3.5\;ms$ . The short-term shear modulus is defined as, ${G\left(0\right)}_{tau}=G_{0tau}=G_{\infty tau}+G_{1tau}=189.83\;MPa$ .

Finally, the matrix is modeled as compressible Neo-Hookean material. The strain energy density function is given as,

$$W=\frac{G_{matrix}}{2}\left(I_{1}-3\right)+\frac{1}{d}{\left(J-1\right)}^{2}$$ (8)

Where, $G_{matrix}$ is initial shear modulus, $I_{1}$ is first deviatoric strain invariant, $d=2/K_{matrix}$ is materials incompressibility parameter and J is determinant of the deformation gradient (Inc., 2021). The effective properties of the matrix come from the combined effect of microfilaments and neurofilaments. Nocodazole is used to disrupt the MT network, and the effective Young’s modulus of axon due to the intact structure of microfilaments and neurofilaments is 1.47 kPa (Ouyang et al., 2013). Considering effective Poisson’s ratio of the axon to be 0.33, the initial shear modulus $\left(G_{matrix}\right)$ and bulk modulus $\left(K_{matrix}\right)$ are input as 0.55 and 1.44 kPa, respectively.

## Composite RVE modeling

**FIGURE 4:**
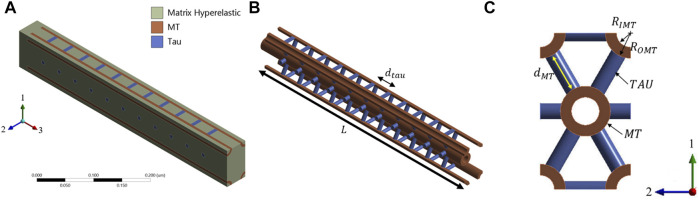
**(A)** Base RVE (RVE-1) for MT and Tau volume fractions of 0.185 and 0.036, respectively. MT is oriented in direction 3. **(B)** MT-Tau crosslinked network is shown with matrix hidden. **(C)** The unit cell of hexagonal array of MT is shown on the 1-2 plane.

The composite RVE of the axon is prepared for the finite element (FEM) micromechanical model. 4 RVEs are modeled by varying the geometric properties. The base RVE is called the RVE-1, and it is modeled for $R_{IMT}=7nm$ , $R_{OMT}=12.5nm$ , $R_{tau}=4nm$ , $d_{MT}=20nm$ , $d_{tau}=40nm$ and L=0.5μm (Figure 4). The estimated volume fraction of MT $\left(\alpha_{MT}\right)$ and Tau $\left(\alpha_{tau}\right)$ are 0.185 and 0.036, respectively, for the RVE-1. Keeping all other parameters fixed, $R_{tau}$ , $d_{tau}$ and $d_{MT}$ are parametrized for RVE-2, RVE-3, and RVE-4, respectively, and the details are summarized in Table 2.

**TABLE 2:** RVE parameters.

# RVE	Varying parameter and value	$\alpha_{\mathrm{MT}}$	$\alpha_{\mathrm{tau}}$
RVE-1	-	0.185	0.036
RVE-2	$R_{tau}=6nm$	0.185	0.085
RVE-3	$d_{tau}=20nm$	0.185	0.071
RVE-4	$d_{MT}=38nm$	0.094	0.036

### Linear viscoelastic theory

To fully characterize the viscoelastic material properties of the axon, we have conducted relaxation tests. The relaxation test is defined as the time response of the stress as the strain remains constant. Since the viscoelastic material response is a combination of both viscous and elastic behaviors, the time-dependent stress is a function of the strain and time as,

σ=f(ϵ,t) (9)

Hereditary integral is used to represent the constitutive relation between the stress and strain under small deformation assumption as,

$$\sigma_{ij}=\int_{0}^{t}C_{ijkl}\left(t-\tau \right)\frac{d\epsilon_{kl}\left(\tau \right)}{d\tau }d\tau \;\;i,j,k,l=1,2,3$$ (10)

In the above equation, τ is a dummy variable and $C_{ijkl}\left(t\right)$ is the relaxation modulus. In the Voigt vector form, Eq. 10 can be represented as (Naik et al., 2008),

$$\left\{\begin{array}{c} \sigma_{11}\left(t\right)\\ \sigma_{22}\left(t\right)\\ \begin{array}{c} \sigma_{33}\left(t\right)\\ \sigma_{23}\left(t\right)\\ \begin{array}{c} \sigma_{13}\left(t\right)\\ \sigma_{12}\left(t\right) \end{array} \end{array} \end{array}\right\}=\int_{0}^{t}\left[\begin{array}{ccc} C_{1111} & C_{1122} & C_{1133}\\ C_{2211} & C_{1122} & C_{1133}\\ \begin{array}{c} C_{3311}\\ C_{2311}\\ \begin{array}{c} C_{1311}\\ C_{1211} \end{array} \end{array} & \begin{array}{c} C_{3322}\\ C_{2322}\\ \begin{array}{c} C_{1322}\\ C_{1222} \end{array} \end{array} & \begin{array}{c} C_{3333}\\ C_{2333}\\ \begin{array}{c} C_{1333}\\ C_{1233} \end{array} \end{array} \end{array}\;\begin{array}{ccc} C_{1123} & C_{1113} & C_{1112}\\ C_{2223} & C_{2213} & C_{2212}\\ \begin{array}{c} C_{3323}\\ C_{2323}\\ \begin{array}{c} C_{1323}\\ C_{1223} \end{array} \end{array} & \begin{array}{c} C_{3313}\\ C_{2313}\\ \begin{array}{c} C_{1313}\\ C_{1213} \end{array} \end{array} & \begin{array}{c} C_{3312}\\ C_{2312}\\ \begin{array}{c} C_{1312}\\ C_{1212} \end{array} \end{array} \end{array}\right]\left\{\begin{array}{c} d\epsilon_{11}\left(\tau \right)/d\tau \\ d\epsilon_{22}\left(\tau \right)/d\tau \\ \begin{array}{c} d\epsilon_{33}\left(\tau \right)/d\tau \\ d\epsilon_{23}\left(\tau \right)/d\tau \\ \begin{array}{c} d\epsilon_{13}\left(\tau \right)/d\tau \\ d\epsilon_{12}\left(\tau \right)/d\tau \end{array} \end{array} \end{array}\right\}d\tau$$ (11)

For an anisotropic material $C_{ijkl}\left(t\right)$ is a 6 × 6 matrix of the relaxation moduli. However, for different material symmetry, the number of the independent relaxation function could vary between 2 and 21. For the axon microstructure, we have three orthogonal planes of symmetry, and therefore the number of the independent relaxation function reduced to 9. In Voigt notation, they are given as,

$$\left\{\begin{array}{c} \sigma_{1}\left(t\right)\\ \sigma_{2}\left(t\right)\\ \begin{array}{c} \sigma_{3}\left(t\right)\\ \sigma_{4}\left(t\right)\\ \begin{array}{c} \sigma_{5}\left(t\right)\\ \sigma_{6}\left(t\right) \end{array} \end{array} \end{array}\right\}=\int_{0}^{t}\left[\begin{array}{ccc} C_{11} & C_{12} & C_{13}\\ C_{21} & C_{22} & C_{23}\\ \begin{array}{c} C_{31}\\ 0\\ \begin{array}{c} 0\\ 0 \end{array} \end{array} & \begin{array}{c} C_{32}\\ 0\\ \begin{array}{c} 0\\ 0 \end{array} \end{array} & \begin{array}{c} C_{33}\\ 0\\ \begin{array}{c} 0\\ 0 \end{array} \end{array} \end{array}\;\begin{array}{ccc} 0 & 0 & 0\\ 0 & 0 & 0\\ \begin{array}{c} 0\\ C_{44}\\ \begin{array}{c} 0\\ 0 \end{array} \end{array} & \begin{array}{c} 0\\ 0\\ \begin{array}{c} C_{55}\\ 0 \end{array} \end{array} & \begin{array}{c} 0\\ 0\\ \begin{array}{c} 0\\ C_{66} \end{array} \end{array} \end{array}\right]\left\{\begin{array}{c} d\epsilon_{1}\left(\tau \right)/d\tau \\ d\epsilon_{2}\left(\tau \right)/d\tau \\ \begin{array}{c} d\epsilon_{3}\left(\tau \right)/d\tau \\ d\epsilon_{4}\left(\tau \right)/d\tau \\ \begin{array}{c} d\epsilon_{5}\left(\tau \right)/d\tau \\ d\epsilon_{6}\left(\tau \right)/d\tau \end{array} \end{array} \end{array}\right\}d\tau$$ (12)

**FIGURE 5:**
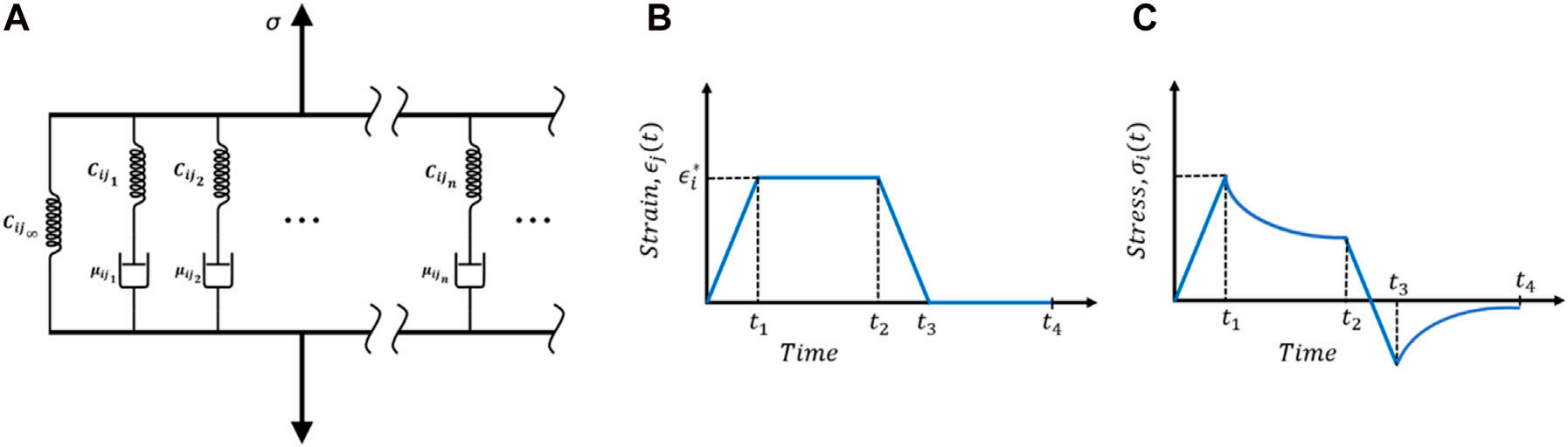
**(A)** General Maxwell viscoelastic model. Typical relaxation test: **(B)** time history of volume-averaged strain and **(C)** stress response.

Due to the tensor symmetry, 9 independent relaxation functions are, $C_{11}$ , $C_{22}$ , $C_{33}$ , $C_{44}$ , $C_{55}$ , $C_{66}$ , $C_{12}=C_{21}$ , $C_{13}=C_{31}$ , and $C_{23}=C_{32}$ . Each of these relaxation functions can be expressed in terms of the Prony series for the General Maxwell model (Figure 5A) given as (Tzikang and Chen, 2000),

$$C_{ij}\left(t\right)={C_{ij}}_{0}\left(1-\overset{n}{\underset{k=1}{\sum }}{h_{ij}}_{k}\left(1-e^{-t/{\tau_{ij}}_{k}}\right)\right)$$ (13)

Where.

The coefficients are defined as, ${h_{ij}}_{k}={C_{ij}}_{k}/{C_{ij}}_{0}$

The relaxation time for each Maxwell element is, ${\tau_{ij}}_{k}={C_{ij}}_{k}/{\mu_{ij}}_{k}$

And the short time modulus is related to the long-term modulus as, ${C_{ij}}_{0}$ = ${C_{ij}}_{\infty }+\overset{n}{\underset{k=1}{\sum }}{C_{ij}}_{k}$ with the subscript *k* refers to the *kth* Maxwell element (see Figure 5A).

Figures 5B,C show a typical relaxation loading condition. Ideally, the strain needed to be step-increased and kept constant for enough long time for the stress to relax. However, in practice, this is not possible; rather, strain is increased in a short period of time until $t_{1}$ and then kept constant for stress to relax until $t_{2}$ . After that, the strain is reduced to zero at $t_{3}$ and the simulation is continued till $t_{4}$ to capture the asymptotic value of the relaxed stress. Therefore, the loading function is given as,

$$\epsilon_{j}\left(t\right)=\left\{ \begin{array}{cc} \epsilon_{j}^{*}t/\left(t_{1}-t_{0}\right) & for\;t_{0}<t\leq t_{1}\\ \epsilon_{j}^{*} & for\;t_{1}<t\leq t_{2}\\ -\epsilon_{j}^{*}t/\left(t_{3}-t_{2}\right) & for\;t_{2}<t\leq t_{3}\\ 0 & for\;t_{3}<t\leq t_{4} \end{array}\right.$$ (14)

$$\frac{d\epsilon_{j}\left(t\right)}{dt}=\left\{ \begin{array}{cc} \epsilon_{j}^{*}/\left(t_{1}-t_{0}\right) & for\;t_{0}<t\leq t_{1}\\ 0 & for\;t_{1}<t\leq t_{2}\\ -\epsilon_{j}^{*}/\left(t_{3}-t_{2}\right) & for\;t_{2}<t\leq t_{3}\\ 0 & for\;t_{3}<t\leq t_{4} \end{array}\right.$$ (15)

In the above two equations j=1,2,3,4,5,6 . Total 6 loading cases are required to fully characterize 9 $C_{ij}\left(t\right)$ of Eq. 12. The simulated data is then used to estimate the volume-averaged time-dependent stress and strain for each time-step for each loading case. The volume-averaged stress and strain is defined as,

$$\sigma_{i}\left(t\right)=\frac{1}{V_{RVE}}\int_{V}{\left[\sigma_{i}\left(t\right)\right]}_{\rho }{dV}_{\rho }$$ (16)

$$\epsilon_{j}\left(t\right)=\frac{1}{V_{RVE}}\int_{V}{\left[\epsilon_{j}\left(t\right)\right]}_{\rho }{dV}_{\rho }$$ (17)

Where, $V_{RVE}$ is the volume of the RVE. In the above two equations, the ${\left[\sigma_{i}\left(t\right)\right]}_{\rho }$ , ${\left[\epsilon_{j}\left(t\right)\right]}_{\rho }$ and ${dV}_{\rho }$ are the stress, strain, and volume, respectively, of the $\rho^{th}$ -element (i.e., mesh element) of the finite element model. For every time step, the instantaneous volume averaged stress-strain constitutive relation is then used to estimate the relaxation function from Eq. 18,

$$\sigma_{i}=\begin{array}{cc} \int_{0}^{t}C_{ij}\left(t-\tau \right)\frac{d\epsilon_{j}\left(\tau \right)}{d\tau }d\tau & i,j=1,2,3,4,5,6 \end{array}$$ (18)

Once the relaxation function is realized, then a nonlinear regression analysis is done to curve fit the coefficients of Eq. 13. For n number of Maxwell elements (spring and damper in series), total 2n+1 coefficients are needed to fit (see Figure 5A).

### Load cases

**FIGURE 6:**
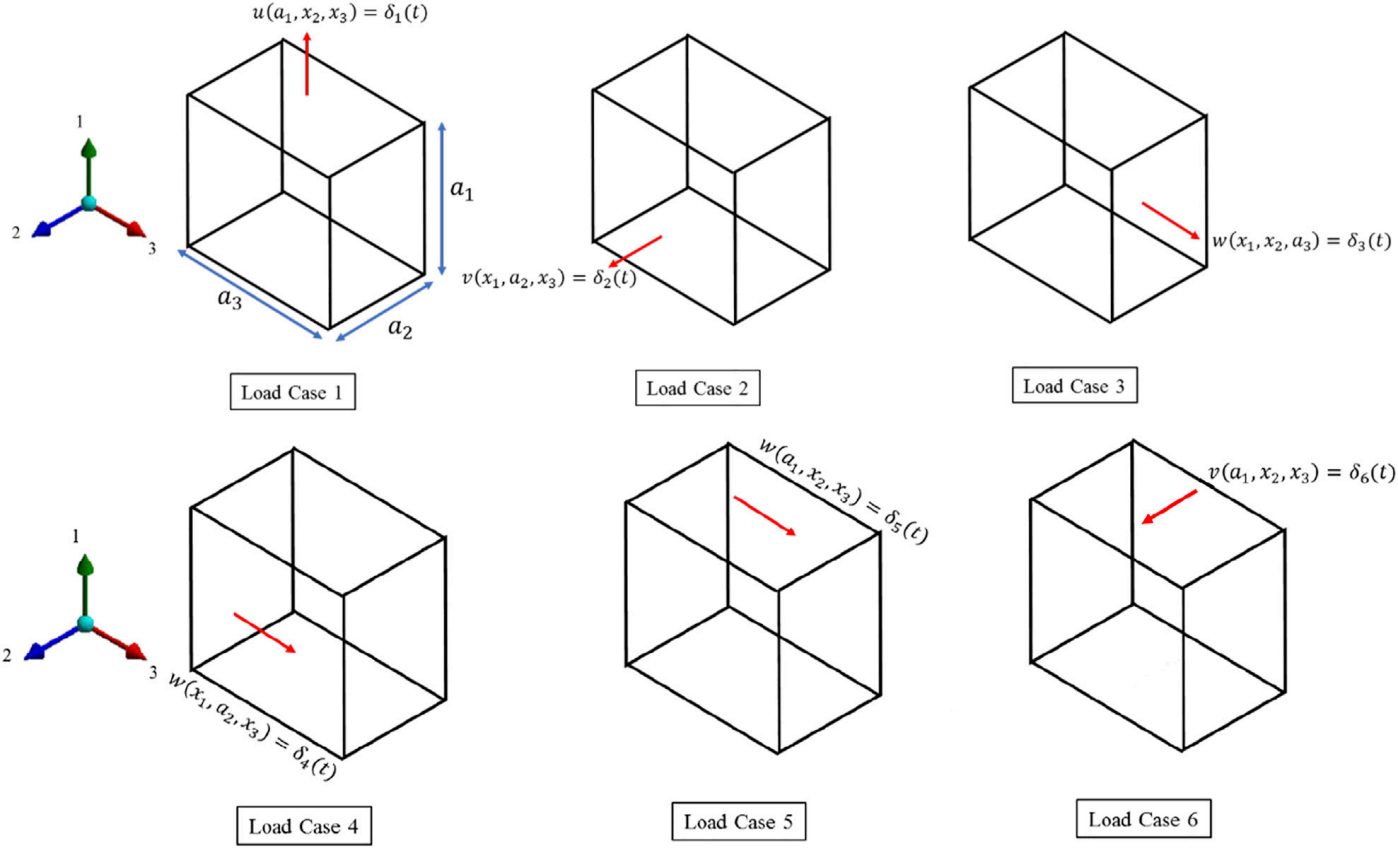
6 load cases for the viscoelastic characterization.

As discussed in the previous section, 6 load cases are required for complete viscoelastic material characterization of the axon RVE. Figures 5B,C show a typical loading function and for all cases, $t_{1}$ , $t_{2}$ , $t_{3}$ and $t_{4}$ are 3 ms , 12 ms , 15 ms, and 40 ms , respectively. Figure 6 shows the deformation boundary condition for the individual load case. We have adopted the composite RVE analysis proposed by Sun et al. (1996) (Sun and Vaidya, 1996b). The first three load cases are for axial loading, while the last three cases impose shear deformation.

#### Load Case 1, 2 and 3

For the first three load cases, the face at $x_{3}=0$ was defined as the symmetric region with displacement boundary condition defined as,

$$face,\;x_{3}=0\;\;w\left(x_{1},x_{2},0\right)=0$$ (19)

In the above equation, $x_{1}$ , $x_{2}$ and $x_{3}$ are principal coordinates, and u , v, and w are displacements, respectively, in the three principal coordinates shown in Figure 6. The displacement boundary conditions for the other five faces for the first three load cases are,

Load Case 1

$$face,\;x_{1}=0\;\;u\left(0,x_{2},x_{3}\right)=0$$

$$face,\;x_{1}=a_{1}\;\;u\left(a_{1},x_{2},x_{3}\right)=\delta_{1}\left(t\right)$$

$$face,\;x_{2}=0\;\;v\left(x_{1},0,x_{3}\right)=0$$ (20)

$$face,\;x_{2}=a_{2}\;\;v\left(x_{1},a_{2},x_{3}\right)=0$$

$$face,\;x_{3}=a_{3}\;\;w\left(x_{1},x_{2},a_{3}\right)=0$$

Load Case 2

$$face,\;x_{1}=0\;\;u\left(0,x_{2},x_{3}\right)=0$$

$$face,\;x_{1}=a_{1}\;\;u\left(a_{1},x_{2},x_{3}\right)=0$$

$$face,\;x_{2}=0\;\;v\left(x_{1},0,x_{3}\right)=0$$ (21)

$$face,\;x_{2}=a_{2}\;\;v\left(x_{1},a_{2},x_{3}\right)=\delta_{2}\left(t\right)$$

$$face,\;x_{3}=a_{3}\;\;w\left(x_{1},x_{2},a_{3}\right)=0$$

Load Case 3

$$face,\;x_{1}=0\;\;u\left(0,x_{2},x_{3}\right)=0$$

$$face,\;x_{1}=a_{1}\;\;u\left(a_{1},x_{2},x_{3}\right)=0$$

$$face,\;x_{2}=0\;\;v\left(x_{1},0,x_{3}\right)=0$$ (22)

$$face,\;x_{2}=a_{2}\;\;v\left(x_{1},a_{2},x_{3}\right)=0$$

$$face,\;x_{3}=a_{3}\;\;w\left(x_{1},x_{2},a_{3}\right)=\delta_{3}\left(t\right)$$

#### Load Case 4,5 and 6

Three shearing deformation is implemented as.

Load Case 4

$$\begin{array}{cc} face,\;x_{1}=0 & u\left(0,x_{2},x_{3}\right)=0\\ face,\;x_{1}=a_{1} & u\left(a_{1},x_{2},x_{3}\right)=0\\ face,\;x_{2}=0 & \begin{array}{c} v\left(x_{1},0,x_{3}\right)=0\\ w\left(x_{1},0,x_{3}\right)=0 \end{array}\\ face,\;x_{2}=a_{2} & \begin{array}{c} v\left(x_{1},a_{2},x_{3}\right)=0\\ w\left(x_{1},a_{2},x_{3}\right)=\delta_{4}\left(t\right) \end{array} \end{array}$$ (23)

Load Case 5

$$\begin{array}{cc} face,\;x_{1}=0 & \begin{array}{c} u\left(0,x_{2},x_{3}\right)=0\\ w\left(0,x_{2},x_{3}\right)=0 \end{array}\\ face,\;x_{1}=a_{1} & \begin{array}{c} u\left(a_{1},x_{2},x_{3}\right)=0\\ w\left(a_{1},x_{2},x_{3}\right)=\delta_{5}\left(t\right) \end{array}\\ face,\;x_{2}=0 & v\left(x_{1},0,x_{3}\right)=0\\ face,\;x_{2}=a_{2} & v\left(x_{1},a_{2},x_{3}\right)=0 \end{array}$$ (24)

Load Case 6

$$\begin{array}{cc} face,\;x_{1}=0 & \begin{array}{c} u\left(0,x_{2},x_{3}\right)=0\\ v\left(0,x_{2},x_{3}\right)=0 \end{array}\\ face,\;x_{1}=a_{1} & \begin{array}{c} u\left(a_{1},x_{2},x_{3}\right)=0\\ v\left(a_{1},x_{2},x_{3}\right)=\delta_{6}\left(t\right) \end{array}\\ face,\;x_{3}=0 & w\left(x_{1},x_{2},0\right)=0\\ face,\;x_{3}=a_{3} & w\left(x_{1},x_{2},a_{3}\right)=0 \end{array}$$ (25)

### Prony series fit to the time-dependent strain

Once we have the simulation data for the volume-averaged stress and strain (Eqs 16, 17) for different load cases, we can utilize a formulation that can be used to fit the Prony series parameters given in Eq. 13. The hereditary integral in Eq. 18 will be used by substituting the strain function (Eqs 14, 15) and the Prony kernel (Eq. 13).

#### Mathematical formulation

For the multiple loading process, the hereditary integral is done for the four steps of loading shown in Figures 5B,C. Considering $\epsilon_{0}=\epsilon \left(0\right)=0$ and $t_{0}=0$ we can derive the formulation in four steps as, Step 1: $0<t\leq t_{1}$

$$\sigma_{i}\left(t\right)=\epsilon_{0}C_{ij}\left(t\right)+\int_{0}^{t}C_{ij}\left(t-\tau \right)\frac{d\epsilon_{j}\left(\tau \right)}{d\tau }d\tau$$

$$=\frac{{C_{ij}}_{0}\epsilon_{i}^{*}}{t_{1}}\left[t-\overset{n}{\underset{k=1}{\sum }}{h_{ij}}_{k}t+\overset{n}{\underset{k=1}{\sum }}{h_{ij}}_{k}{\tau_{ij}}_{k}-\overset{n}{\underset{k=1}{\sum }}{h_{ij}}_{k}{\tau_{ij}}_{k}e^{-t/{\tau_{ij}}_{k}}\right]$$ (26)

Step 2: $t_{1}<t\leq t_{2}$

$$\sigma_{i}\left(t\right)=\epsilon_{0}C_{ij}\left(t\right)+\int_{0}^{t_{1}-}C_{ij}\left(t-\tau \right)\frac{d\epsilon_{j}\left(\tau \right)}{d\tau }d\tau +\int_{t_{1}+}^{t}C_{ij}\left(t-\tau \right)\frac{d\epsilon_{j}\left(\tau \right)}{d\tau }d\tau$$

$$=\frac{{C_{ij}}_{0}\epsilon_{i}^{*}}{t_{1}}\left[t_{1}-\overset{n}{\underset{k=1}{\sum }}{h_{ij}}_{k}t_{1}+\overset{n}{\underset{k=1}{\sum }}{h_{ij}}_{k}{\tau_{ij}}_{k}e^{-\left(t-t_{1}\right)/{\tau_{ij}}_{k}}-\overset{n}{\underset{k=1}{\sum }}{h_{ij}}_{k}{\tau_{ij}}_{k}e^{-t/{\tau_{ij}}_{k}}\right]$$ (27)

Step 3: $t_{2}<t\leq t_{3}$

$$\sigma_{i}\left(t\right)=\epsilon_{0}C_{ij}\left(t\right)+\int_{0}^{t_{1}-}C_{ij}\left(t-\tau \right)\frac{d\epsilon_{j}\left(\tau \right)}{d\tau }d\tau +\int_{t_{1}+}^{t_{2}-}C_{ij}\left(t-\tau \right)\frac{d\epsilon_{j}\left(\tau \right)}{d\tau }d\tau +\int_{t_{2}+}^{t}C_{ij}\left(t-\tau \right)\frac{d\epsilon_{j}\left(\tau \right)}{d\tau }d\tau$$

$$\begin{array}{ll} = & \frac{{C_{ij}}_{0}\epsilon_{i}^{*}}{t_{1}}\left[t_{1}-\overset{n}{\underset{k=1}{\sum }}{h_{ij}}_{k}t_{1}+\overset{n}{\underset{k=1}{\sum }}{h_{ij}}_{k}{\tau_{ij}}_{k}e^{-\left(t-t_{1}\right)/{\tau_{ij}}_{k}}-\overset{n}{\underset{k=1}{\sum }}{h_{ij}}_{k}{\tau_{ij}}_{k}e^{-t/{\tau_{ij}}_{k}}\right]\\ & -\frac{{C_{ij}}_{0}\epsilon_{i}^{*}}{\left(t_{3}-t_{2}\right)}\left[t-\overset{n}{\underset{k=1}{\sum }}{h_{ij}}_{k}t+\overset{n}{\underset{k=1}{\sum }}{h_{ij}}_{k}{\tau_{ij}}_{k}-t_{2}+\overset{n}{\underset{k=1}{\sum }}{h_{ij}}_{k}t_{2}-\overset{n}{\underset{k=1}{\sum }}{h_{ij}}_{k}{\tau_{ij}}_{k}e^{-\left(t-t_{2}\right)/{\tau_{ij}}_{k}}\right] \end{array}$$ (28)

Step 4: $t_{3}<t\leq t_{4}$

$$\sigma_{i}\left(t\right)=\epsilon_{0}C_{ij}\left(t\right)+\int_{0}^{t_{1}-}C_{ij}\left(t-\tau \right)\frac{d\epsilon_{j}\left(\tau \right)}{d\tau }d\tau +\int_{t_{1}+}^{t_{2}-}C_{ij}\left(t-\tau \right)\frac{d\epsilon_{j}\left(\tau \right)}{d\tau }d\tau +\int_{t_{2}+}^{t_{3}-}C_{ij}\left(t-\tau \right)\frac{d\epsilon_{j}\left(\tau \right)}{d\tau }d\tau +\int_{t_{3}+}^{t}C_{ij}\left(t-\tau \right)\frac{d\epsilon_{j}\left(\tau \right)}{d\tau }d\tau$$

$$\begin{array}{ll} = & \frac{{C_{ij}}_{0}\epsilon_{i}^{*}}{t_{1}}\left[t_{1}-\overset{n}{\underset{k=1}{\sum }}{h_{ij}}_{k}t_{1}+\overset{n}{\underset{k=1}{\sum }}{h_{ij}}_{k}{\tau_{ij}}_{k}e^{-\left(t-t_{1}\right)/{\tau_{ij}}_{k}}-\overset{n}{\underset{k=1}{\sum }}{h_{ij}}_{k}{\tau_{ij}}_{k}e^{-t/{\tau_{ij}}_{k}}\right]\\ & -\frac{{C_{ij}}_{0}\epsilon_{i}^{*}}{\left(t_{3}-t_{2}\right)}\left[t_{3}-\overset{n}{\underset{k=1}{\sum }}{h_{ij}}_{k}t_{3}+\overset{n}{\underset{k=1}{\sum }}{h_{ij}}_{k}{\tau_{ij}}_{k}e^{-\left(t-t_{3}\right)/{\tau_{ij}}_{k}}-t_{2}+\overset{n}{\underset{k=1}{\sum }}{h_{ij}}_{k}t_{2}-\overset{n}{\underset{k=1}{\sum }}{h_{ij}}_{k}{\tau_{ij}}_{k}e^{-\left(t-t_{2}\right)/{\tau_{ij}}_{k}}\right] \end{array}$$ (29)

We have found that n=2 (two Mx elements in Figure 5A) can provide a good fit to the data, hence total (2n+1=) 5 parameters ( ${C_{ij}}_{0},\;{h_{ij}}_{1},{h_{ij}}_{2},{\tau_{ij}}_{1}\;and\;{\tau_{ij}}_{2}$ ) are reported for each relaxation function $\left(C_{ij}\left(t\right)\right)$ .

#### Nonlinear regression for the curve fitting

The Prony series coefficients defined in Eqs 26–29 will be estimated using the nonlinear regression method. The Marquardt-Levenberg method has been implemented for the data fit (Press et al., 1989). The error function $\left(\chi^{2}\right)$ is minimized for unknown constant coefficients (C) in every iteration and given as (Gavin, 2019b),

$${\chi \left(C\right)}^{2}=\overset{N_{d}}{\underset{i=1}{\sum }}{\left[\frac{y_{i}-y\left(x_{i};C\right)}{{\sigma_{SD}}_{i}}\right]}^{2}$$ (30)

Where, $N_{d}$ is the number of data points, the simulated stress, and strain data points are $y_{i}$ and $x_{i}$ , respectively. Function $y\left(x_{i};C\right)$ is given by Eqs 26–29 for coefficient vector, $C={\left\{{C_{ij}}_{0},\;{h_{ij}}_{1},{h_{ij}}_{2},{\tau_{ij}}_{1},{\tau_{ij}}_{2}\right\}}^{T}$ . For the $i^{th}$ data point the standard deviation is given by ${\sigma_{SD}}_{i}$ . Initial values of vector C are defined first, and then at each iteration the ${\chi \left(C\right)}^{2}$ is minimized. An improved C is estimated until ${\chi \left(C\right)}^{2}$ reaches a minimum and does not change. The thermodynamic constraint for each coefficient is defined as (Tzikang and Chen, 2000),

$${C_{ij}}_{0}>0,\;{h_{ij}}_{k}\geq 0,\sum {h_{ij}}_{k}\leq 1\;and\;{\tau_{ij}}_{k}\geq 0$$ (31)

## Results and discussion

### Viscoelastic characterization of axon

A total of 30 simulations have been carried out for 4 different RVEs. In the first 6 simulations, the Young’s Modulus of MT is set to 1.5 GPa and then increased to 1.9 GPa for another set of 6 simulations with RVE-1. Then rest of the 18 simulations are performed for three RVEs (RVE-2, RVE-3, and RVE-4) with $E_{MT}=1.5\;GPa$ . Results from a total of 30 simulations are discussed here.

#### Load case 1, 2 and 3

**FIGURE 7:**
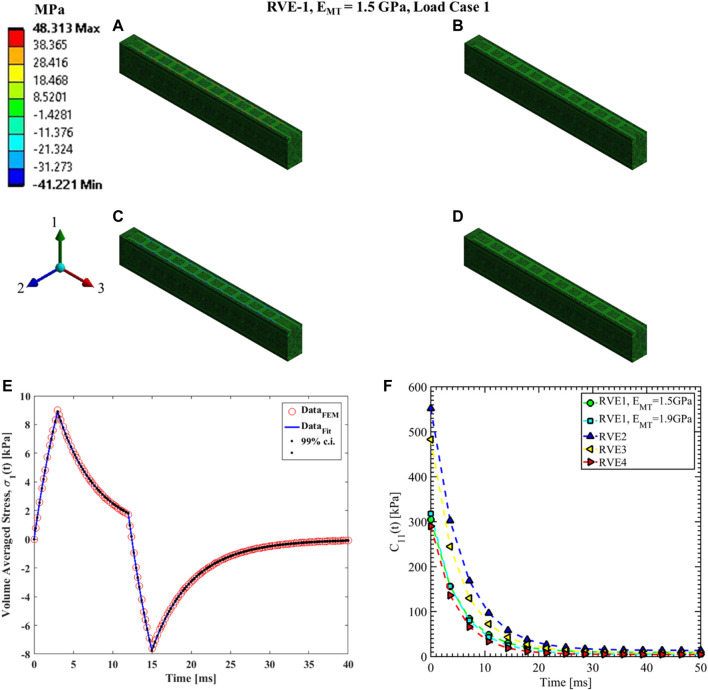
Contour plot of the element normal stress in direction 1 ( ${\left[\sigma_{1}\left(t\right)\right]}_{\rho }$ from Eq. 16). $C_{11}\left(t\right)$ estimation for the RVE-1 with $E_{MT}=1.5\;GPa$ by imposing the load case 1. **(A)** $t_{1}=3ms$ , $\delta_{1}\left(t_{1}\right)=3nm$ **(B)** $t_{2}=12ms$ , $\delta_{1}\left(t_{2}\right)=3nm$ **(C)** $t_{3}=15ms$ , $\delta_{1}\left(t_{3}\right)=0nm$ **(D)** $t_{4}=40ms$ , $\delta_{1}\left(t_{4}\right)=0nm$ . **(E)** Nonlinear regression data fit with 99% confidence interval (c.i.) is shown for volume averaged normal stress in direction 1 for RVE-1, $E_{MT}=1.5\;GPa$ for load case 1. **(F)** Comparison of $C_{11}\left(t\right)$ for different RVEs.

**FIGURE 8:**
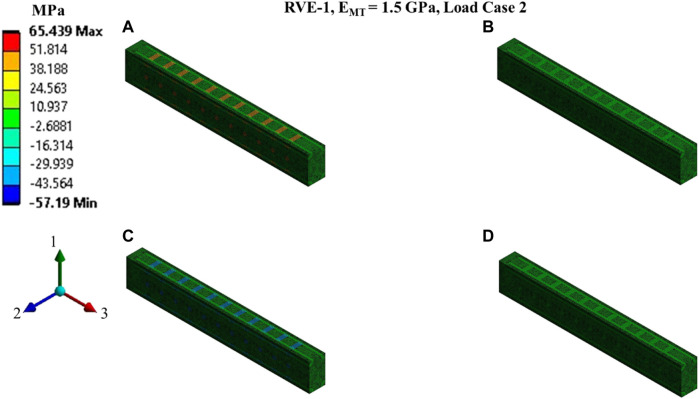
Contour plot of the element normal stress in direction 2 ( ${\left[\sigma_{2}\left(t\right)\right]}_{\rho }$ from Eq. 16). $C_{12}\left(t\right)$ and $C_{22}\left(t\right)$ estimation for the RVE-1 with $E_{MT}=1.5\;GPa$ by imposing the load case 2. **(A)** $t_{1}=3ms$ , $\delta_{2}\left(t_{1}\right)=3nm$ **(B)** $t_{2}=12ms$ , $\delta_{2}\left(t_{2}\right)=3nm$ **(C)** $t_{3}=15ms$ , $\delta_{2}\left(t_{3}\right)=0nm$ **(D)** $t_{4}=40ms$ , $\delta_{2}\left(t_{4}\right)=0nm$ .

**FIGURE 9:**
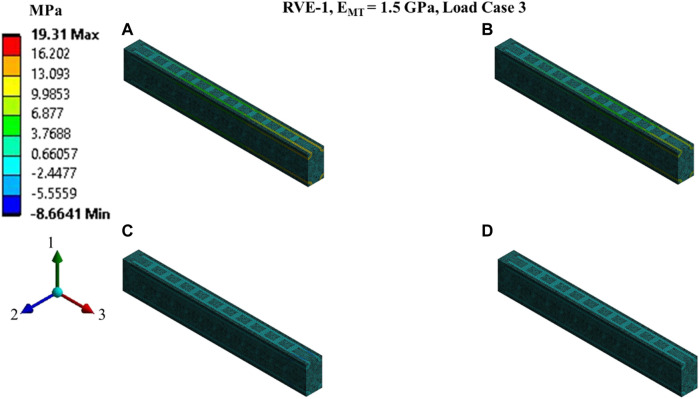
Contour plot of the element normal stress in direction 3 ( ${\left[\sigma_{3}\left(t\right)\right]}_{\rho }$ from Eq. 16). $C_{13}\left(t\right)$ , $C_{23}\left(t\right)$ and $C_{33}\left(t\right)$ estimation for the RVE-1 with $E_{MT}=1.5\;GPa$ by imposing the load case 3. **(A)** $t_{1}=3ms$ , $\delta_{3}\left(t_{1}\right)=3nm$ **(B)** $t_{2}=12ms$ , $\delta_{3}\left(t_{2}\right)=3nm$ **(C)** $t_{3}=15ms$ , $\delta_{3}\left(t_{3}\right)=0nm$ **(D)** $t_{4}=40ms$ , $\delta_{3}\left(t_{4}\right)=0nm$ .

The coefficients of the Prony series for the relaxation function $C_{11}\left(t\right)$ are estimated from load case 1 for the different RVEs. $C_{12}\left(t\right)$ and $C_{22}\left(t\right)$ are estimated from load case 2 while $C_{13}\left(t\right)$ , $C_{23}\left(t\right)$ and $C_{33}\left(t\right)$ are estimated using load case 3. Representative contour plots of elemental stress for the three load cases are given in Figures 7, 8, 9. In those figures, we have shown plots for RVE-1 with $E_{MT}=1.5\;GPa$ .

**FIGURE 10:**
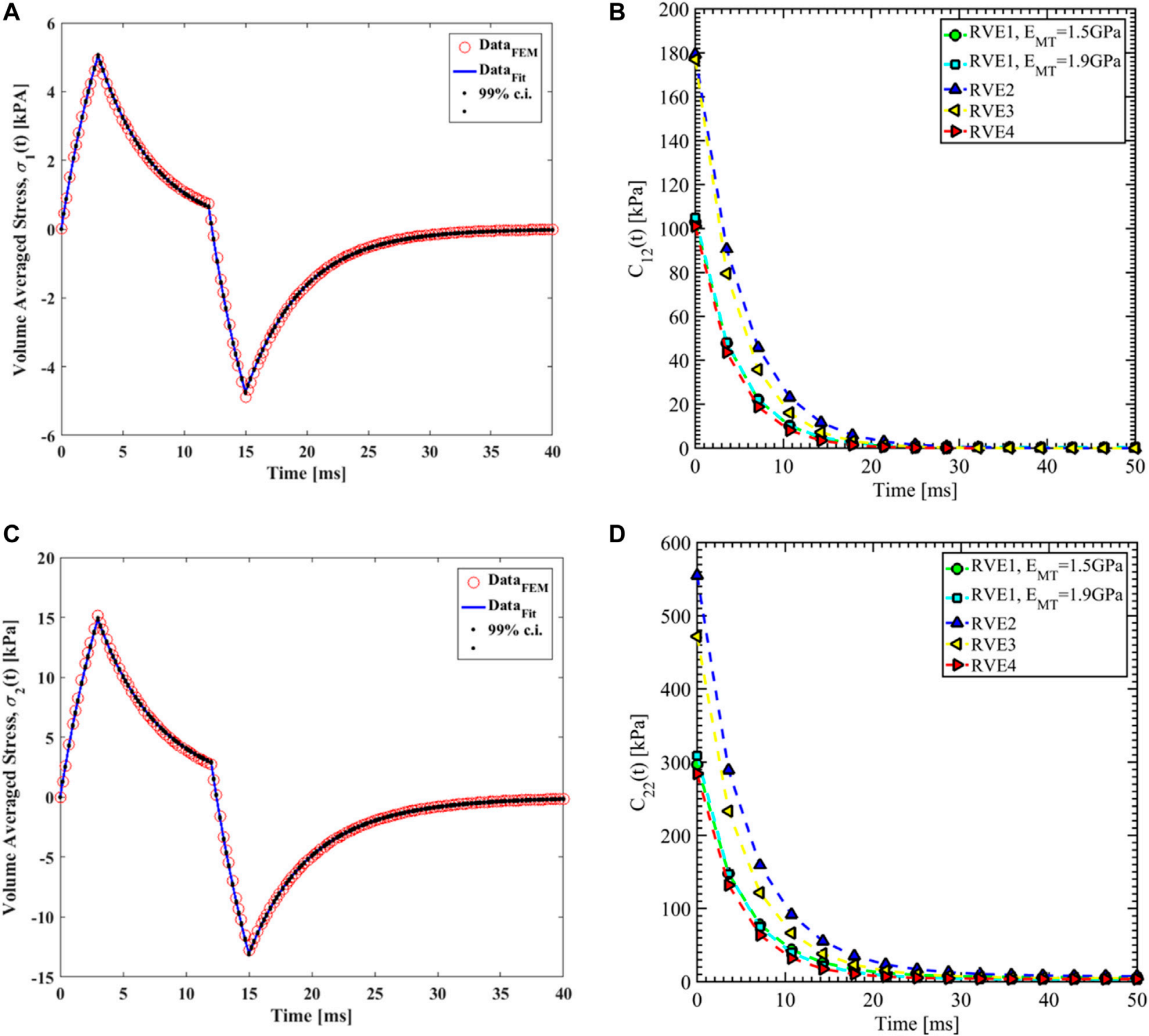
**(A)** Nonlinear regression data fit with 99% confidence interval (c.i.) is shown for volume averaged normal stress in direction 1 for RVE-1, $E_{MT}=1.5\;GPa$ for load case 2. **(B)** Comparison of $C_{12}\left(t\right)$ for different RVEs. **(C)** Nonlinear regression data fit with 99% confidence interval (c.i.) is shown for volume averaged normal stress in direction 2 for RVE-1, $E_{MT}=1.5\;GPa$ for load case 2. **(D)** Comparison of $C_{22}\left(t\right)$ for different RVEs.

**FIGURE 11:**
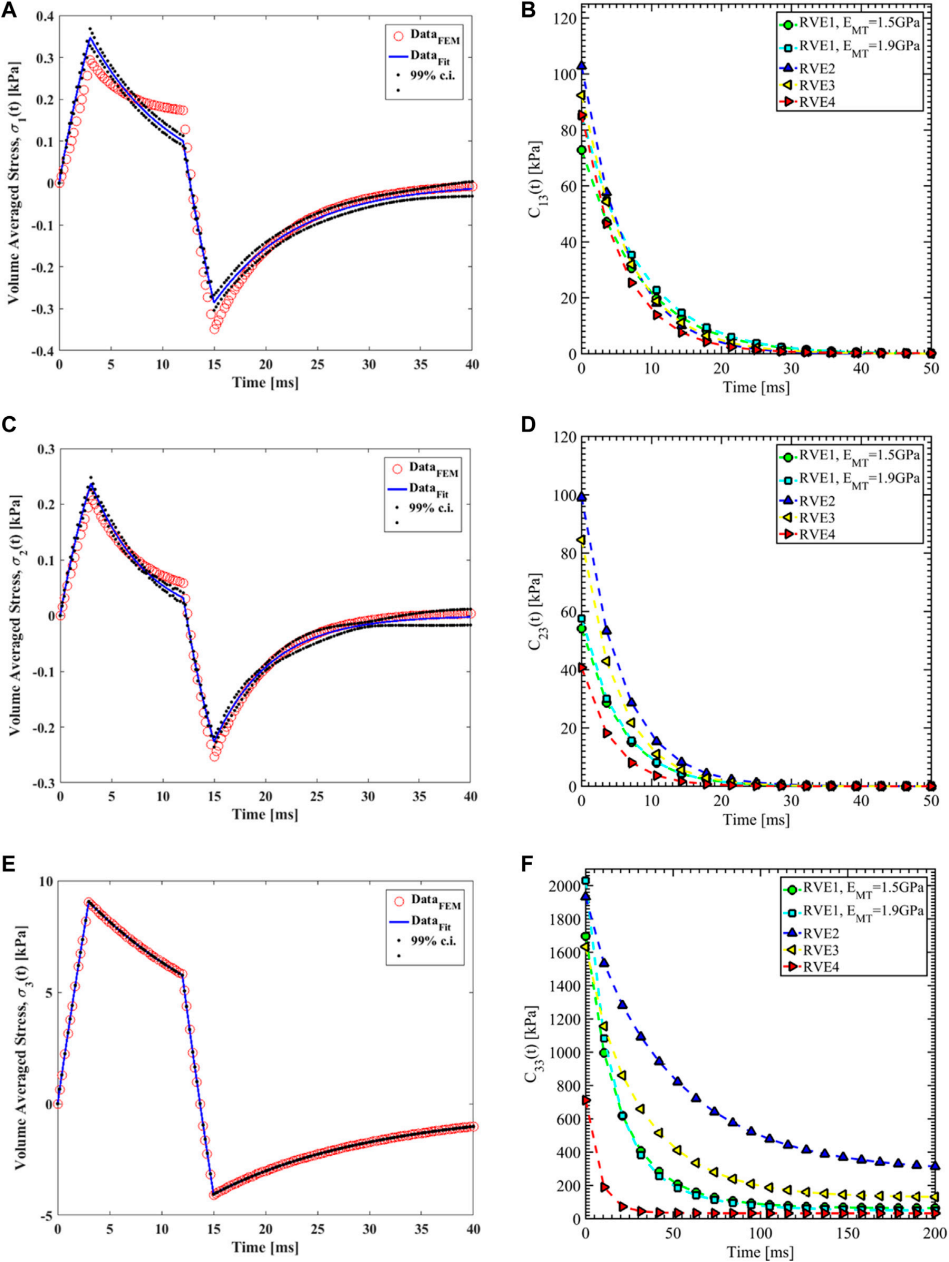
**(A)** Nonlinear regression data fit with 99% confidence interval (c.i.) is shown for volume averaged normal stress in direction 1 for RVE-1, $E_{MT}=1.5\;GPa$ for load case 3. **(B)** Comparison of $C_{13}\left(t\right)$ for different RVEs. **(C)** Nonlinear regression data fit with 99% confidence interval (c.i.) is shown for volume averaged normal stress in direction 2 for RVE-1, $E_{MT}=1.5\;GPa$ for load case 3. **(D)** Comparison of $C_{23}\left(t\right)$ for different RVEs. **(E)** Nonlinear regression data fit with 99% confidence interval (c.i.) is shown for volume averaged normal stress in direction 3 for RVE-1, $E_{MT}=1.5\;GPa$ for load case 3. **(F)** Comparison of $C_{33}\left(t\right)$ for different RVEs.

Nonlinear regression data fit for the Prony series coefficients using the volume-averaged stress from FEM simulations and Eqs 26–29 are shown in Figures 7E, 10A,C, 11A,C,E for load case 1 to 3, respectively. In those figures, Data_FEM_ and Data_Fit_ corresponds to $y_{i}$ and $y\left(x_{i};C\right)$ in Eq. 30, respectively. Figures 7F, 10B,D, 11B,D,F show the comparison of $C_{ij}\left(t\right)$ for different RVEs for the first three cases.

Since the orientation of the MT is in direction 3, Young’s modulus of MT has no significant effects on the relaxation functions in the transverse plane ( $C_{11}\left(t\right),C_{12}\left(t\right),C_{22}\left(t\right),C_{13}\left(t\right)\;and\;C_{23}\left(t\right)$ ). Increasing Young’s modulus of MT showed increased short-term relaxation modulus in the direction 3 ( ${C_{33}}_{0}$ ). The increasing Young’s modulus does not affect the relaxation time for $C_{33}\left(t\right)$ as well.

The volume fraction of Tau ( $\alpha_{tau}$ ) is increased in RVE-2 and RVE-3 while the volume fraction of MT ( $\alpha_{MT}$ ) is kept constant as the base RVE (i.e., RVE-1). In RVE-2, we increased the $\alpha_{tau}$ by increasing the Tau radius to 6 nm, while in RVE-3, we increased the Tau density by reducing the Tau spacing to 20 nm. In the first three load cases, increasing $\alpha_{tau}$ has shown increased short-term relaxation modulus ( ${C_{ij}}_{0}$ ) for all relaxation functions ( $C_{ij}\left(t\right)$ ). There is no effect on the relaxation time except for $C_{33}\left(t\right)$ where RVE-2 showed a longer relaxation time compared to RVE-3.

In RVE-4, the $\alpha_{MT}$ is reduced by increasing the end-to-end distance between the MTs to 38 nm and $\alpha_{tau}$ is kept constant to the value of base RVE-1. Again, the effect of the $\alpha_{MT}$ is insignificant in the transverse plan as expected. However, decreasing $\alpha_{MT}$ has the most interesting effect on the $C_{33}\left(t\right)$ as the short-term relaxation modulus ( ${C_{33}}_{0}$ ) is almost reduced to half, and the relaxation time is the fastest compared to other RVEs. Overall, the stiffness of the axon in direction 3 is 5–7 times higher than the stiffness in directions 1 and 2. The Prony series coefficients are tabulated in Table 3 with the mean and standard error associated with the 5 observations (2 for RVE-1 and 3 for RVE-2, RVE-3, and RVE-4).

**TABLE 3:** Prony series coefficients for all $C_{ij}\left(t\right)$ .

$C_{ij}\left(t\right)$	${C_{ij}}_{0}\;\left[kPa\right]$	${h_{ij}}_{1}$	${h_{ij}}_{2}$	${\tau_{ij}}_{1}\left[ms\right]$	${\tau_{ij}}_{2}\;\left[ms\right]$
$C_{11}$	389.60 ± 47.90	0.43 ± 0.02	0.55 ± 0.02	4.54 ± 0.40	5.66 ± 0.40
$C_{12}$	133.09 ± 16.48	0.35 ± 0.05	0.65 ± 0.05	4.63 ± 0.14	4.64 ± 0.15
$C_{22}$	383.44 ± 49.03	0.31 ± 0.01	0.68 ± 0.01	3.32 ± 0.05	6.01 ± 0.24
$C_{13}$	87.68 ± 4.40	0.41 ± 0.05	0.59 ± 0.05	7.02 ± 0.46	7.06 ± 0.43
$C_{23}$	67.27 ± 9.58	0.43 ± 0.03	0.57 ± 0.03	5.37 ± 0.15	5.27 ± 0.25
$C_{33}$	1601.38 ± 209.24	0.19 ± 0.07	0.75 ± 0.06	19.27 ± 5.08	23.84 ± 7.49
$C_{44}$	68.68 ± 16.85	0.22 ± 0.07	0.76 ± 0.07	7.70 ± 0.95	4.77 ± 0.23
$C_{55}$	48.46 ± 13.35	0.18 ± 0.07	0.80 ± 0.07	8.80 ± 0.68	4.74 ± 0.19
$C_{66}$	80.55 ± 5.20	0.36 ± 0.04	0.62 ± 0.04	5.91 ± 0.51	9.29 ± 1.18

#### Load case 4, 5 and 6

**FIGURE 12:**
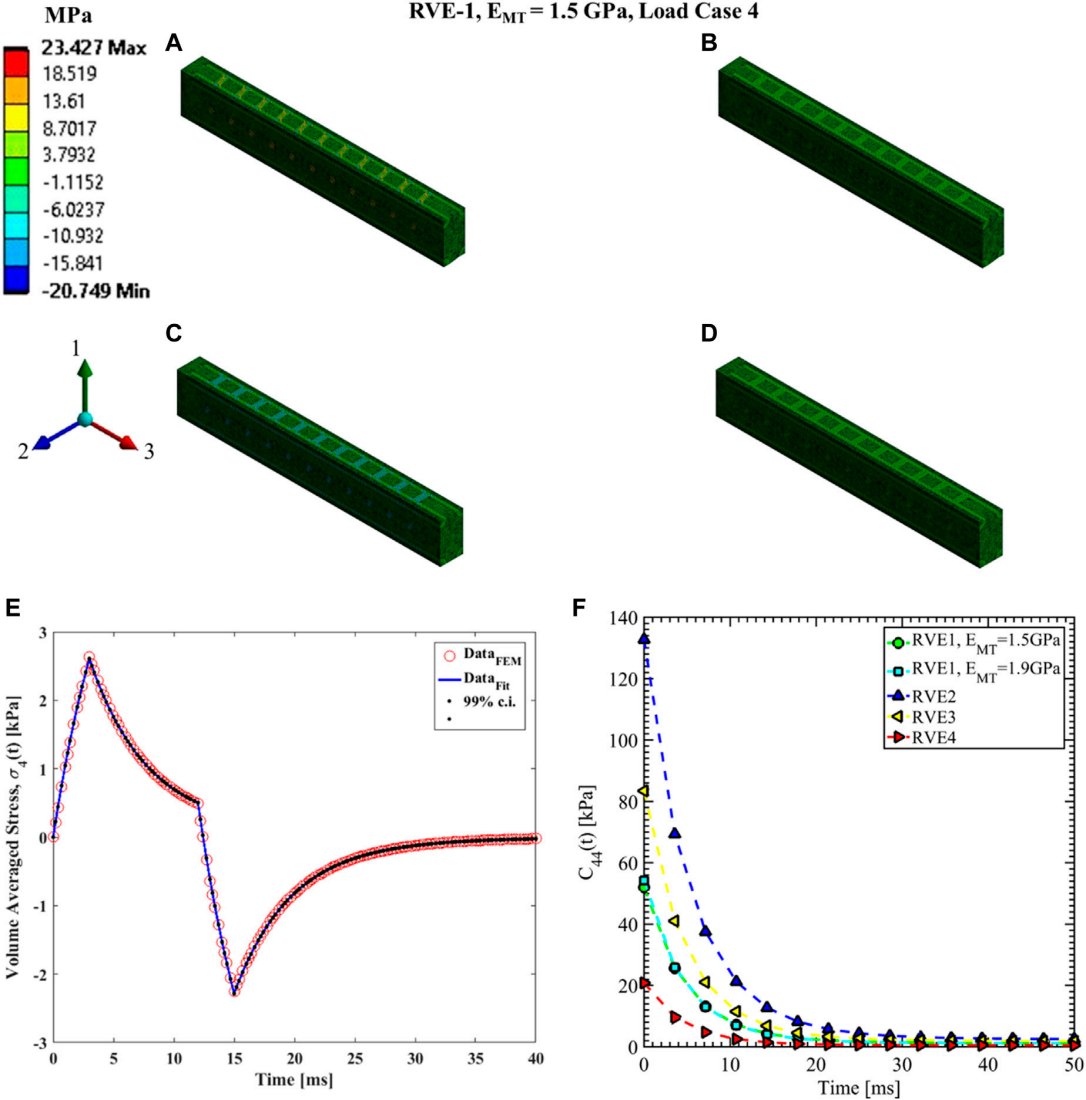
Contour plot of the element shear stress in directions 2 and 3 ( ${\left[\sigma_{4}\left(t\right)\right]}_{\rho }$ from Eq. 16). $C_{44}\left(t\right)$ estimation for the RVE-1 with $E_{MT}=1.5\;GPa$ by imposing the load case 4. **(A)** $t_{1}=3ms$ , $\delta_{4}\left(t_{1}\right)=3nm$ **(B)** $t_{2}=12ms$ , $\delta_{4}\left(t_{2}\right)=3nm$ **(C)** $t_{3}=15ms$ , $\delta_{4}\left(t_{3}\right)=0nm$ **(D)** $t_{4}=40ms$ , $\delta_{4}\left(t_{4}\right)=0nm$ . **(E)** Nonlinear regression data fit with 99% confidence interval (c.i.) is shown for volume averaged shear stress in directions 2 and 3 for RVE-1, $E_{MT}=1.5\;GPa$ for load case 4. **(F)** Comparison of $C_{44}\left(t\right)$ for different RVEs.

**FIGURE 13:**
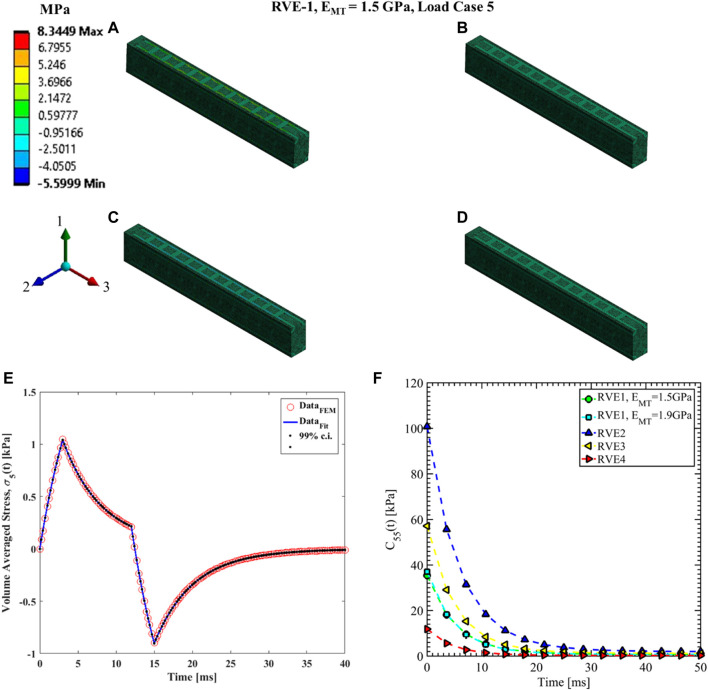
Contour plot of the element shear stress in directions 1 and 3 ( ${\left[\sigma_{5}\left(t\right)\right]}_{\rho }$ from Eq. 16). $C_{55}\left(t\right)$ estimation for the RVE-1 with $E_{MT}=1.5\;GPa$ by imposing the load case 5. **(A)** $t_{1}=3ms$ , $\delta_{5}\left(t_{1}\right)=3nm$ **(B)** $t_{2}=12ms$ , $\delta_{5}\left(t_{2}\right)=3nm$ **(C)** $t_{3}=15ms$ , $\delta_{5}\left(t_{3}\right)=0nm$ **(D)** $t_{4}=40ms$ , $\delta_{5}\left(t_{4}\right)=0nm$ . **(E)** Nonlinear regression data fit with 99% confidence interval (c.i.) is shown for volume averaged shear stress in directions 1 and 3 for RVE-1, $E_{MT}=1.5\;GPa$ for load case 5. **(F)** Comparison of $C_{55}\left(t\right)$ for different RVEs.

**FIGURE 14:**
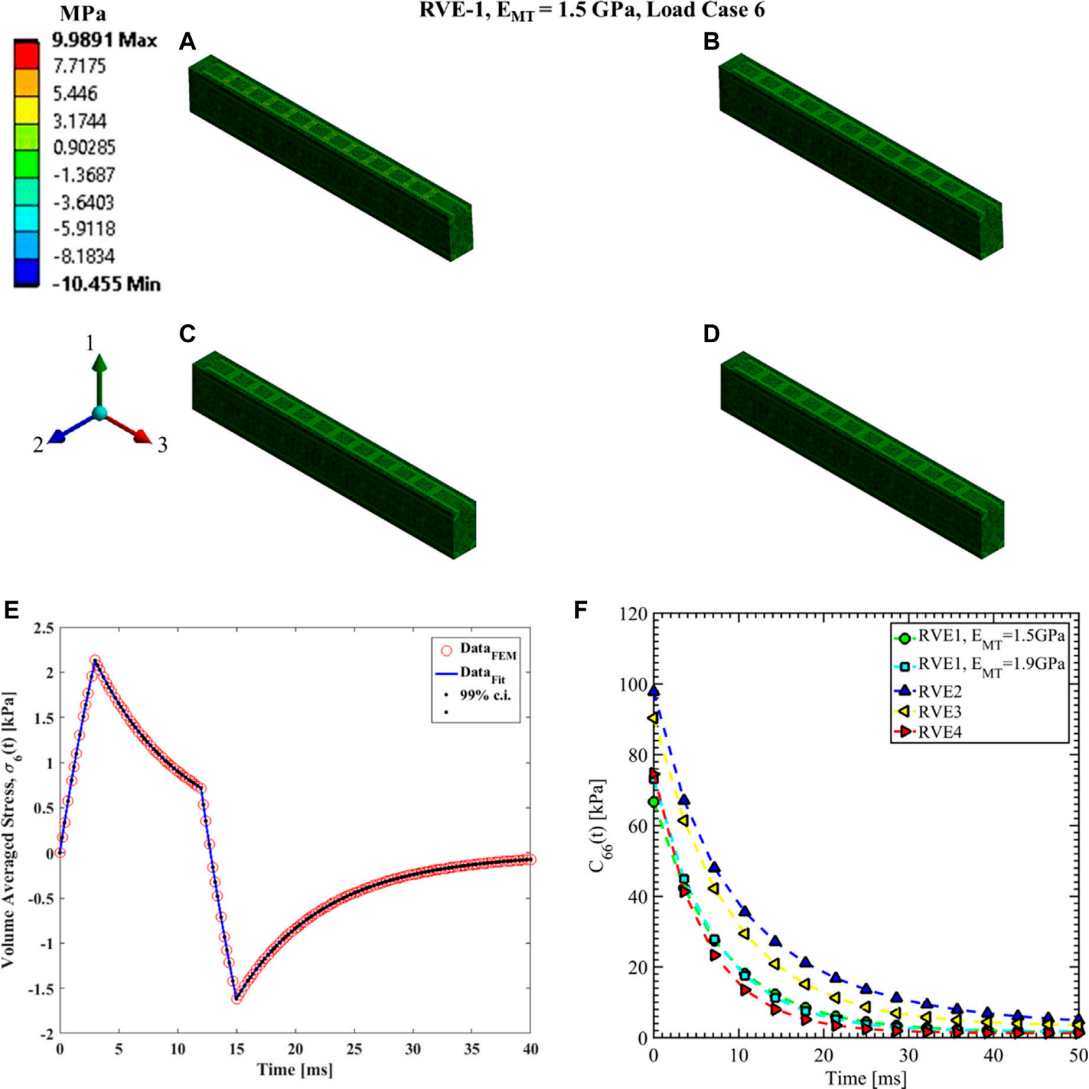
Contour plot of the element shear stress in directions 1 and 2 ( ${\left[\sigma_{6}\left(t\right)\right]}_{\rho }$ from Eq. 16). $C_{66}\left(t\right)$ estimation for the RVE-1 with $E_{MT}=1.5\;GPa$ by imposing the load case 6. **(A)** $t_{1}=3ms$ , $\delta_{6}\left(t_{1}\right)=3nm$ **(B)** $t_{2}=12ms$ , $\delta_{6}\left(t_{2}\right)=3nm$ **(C)** $t_{3}=15ms$ , $\delta_{6}\left(t_{3}\right)=0nm$ **(D)** $t_{4}=40ms$ , $\delta_{6}\left(t_{4}\right)=0nm$ . **(E)** Nonlinear regression data fit with 99% confidence interval (c.i.) is shown for volume averaged shear stress in directions 1 and 2 for RVE-1, $E_{MT}=1.5\;GPa$ for load case 6. **(F)** Comparison of $C_{66}\left(t\right)$ for different RVEs.

The shear relaxation functions are estimated using load cases 4, 5, and 6. Figures 12–14 show the elemental shear stress contour plots for the mentioned load cases on the RVE-1. Nonlinear regression data fit shown in Figures 12E, 13E, 14E, while the comparison of the relaxation function is shown in Figures 12F, 13F, 14F, respectively. Young’s modulus of MT has no significant effects on the shear relaxation functions. As we have seen earlier, the increasing volume fraction of Tau increases the short-term modulus in RVE-2 and RVE-3, respectively. Similarly, decreasing volume fraction of MT showed lower short-term modulus and short relaxation time. From Table 3, it is evident that shearing relaxation functions ( $C_{44}\left(t\right)$ , $C_{55}\left(t\right)$ and $C_{66}\left(t\right)$ ) have lower stiffness compared to the normal relaxation functions ( $C_{11}\left(t\right)$ , $C_{22}\left(t\right)$ and $C_{33}\left(t\right)$ ). This is reflected as well in the macroscale modulus of the brain tissue. The bulk modulus of white matter and gray matter is in the Gigapascal range (∼2 GPa), while the short-term shear modulus is in the kilopascal range (∼40 kPa) (Zhang et al., 2004).

**FIGURE 15:**
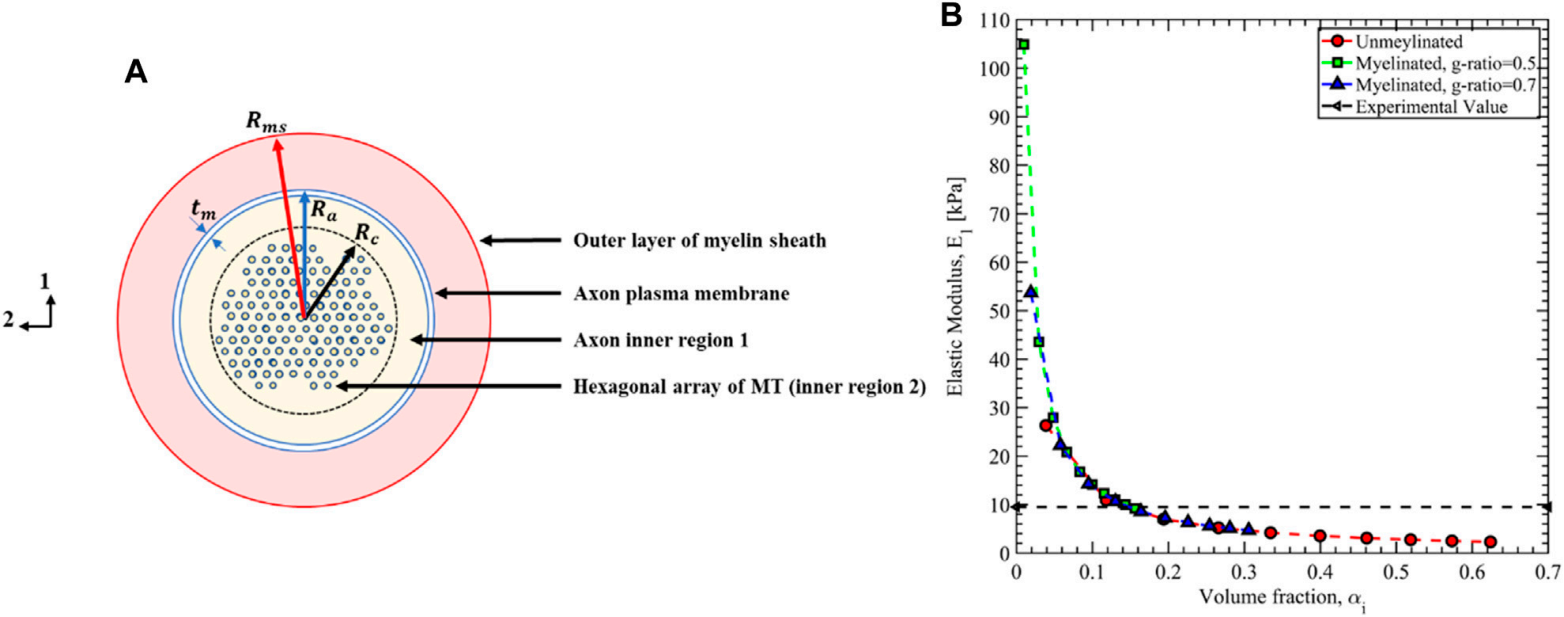
**(A)** Cross-sectional view of the axon microstructure. **(B)** The axon’s effective transverse elastic modulus is estimated using the inverse rule of mixture based on the composite theory.

In Table 3, we have summarized the mean value of the Prony series coefficients for all 5 observations along with the standard error defined as $\frac{\sigma }{\sqrt{N}}$ where σ is the standard deviation and N is the number of observations. To the best of our knowledge, this is the first attempt to fully characterize the viscoelastic responses of the axon cytoskeletal network. Therefore, there is no data available to validate or compare the values tabulated in Table 3 directly. However, there were numerous attempts to estimate the mechanical properties of axons. In Figure 15A, we have drawn the cross-sectional organization of the axon in the transverse plane (e.g., directions 1 and 2). The red outer layer is the myelin sheath, an extension of the lipid bilayer of the oligodendrocytes in the central nervous system (CNS) or the Schwann cells in the peripheral nervous system (PNS). The myelin sheath is typical of the white matter tissue region and does not exist in the gray matter region. The bulk modulus $\left(K_{ms}\right)$ and the Poisson’s ratio $\left(\nu_{ms}\right)$ of the lipid bilayer are 54 MPa and 0.11∼0.44 , respectively (Jadidi et al., 2014), (Ayton et al., 2002). The radius of the axon fiber (with myelin sheath) is defined as $R_{ms}$ . Virtually it is challenging to separate the outer layer of the myelin sheath as sheaths from neighboring axons packed together (Lee et al., 2019). Several studies have shown that the *g*-ratio, defined as the ratio between the axon radius $\left(R_{a}\right)$ and sheath radius $\left(R_{ms}\right)$ , varied between 0.5 and 0.7 (Lee et al., 2019), (Duval et al., 2019). Just beneath the myelin sheath, there is an axon membrane skeleton made of Actin rings connected by Spectrin tetramers with the thickness $\left(t_{m}\right)$ of 10 nm (Zhang et al., 2017), (Xu et al., 1979). The inner-axonal space (IAS) is shown with the yellow shaded area in Figure 15A, with the radius of the IAS defined as $R_{a}$ . In the pyramidal neurons, the cytoskeletal network of MT and Tau is located far from the axon membrane, and hence we defined two inner regions and separated them with the black dotted circle of radius $R_{c}$ . The thickness $\left(R_{a}-R_{c}\right)$ of the inner region 1 can typically extend up to 200 nm (Peters et al., 1968), (Leterrier, 2016). Since both $R_{ms}$ and $R_{c}$ are directly related to the $R_{a}$ by *g*-ratio and axon inner region thickness, respectively, we can estimate their values by carefully choosing the axon radius ( $R_{a}$ ). Ouyang et al. (2013) used atomic force microscopy (AFM) to characterize the transverse elastic modulus (e.g., $E_{1}\;or\;E_{2}$ ) of the unmyelinated axon by separately disrupting the individual cytoskeletal components (Ouyang et al., 2013). They reported axon radius to be 0.5 µm and estimated the transverse elastic modulus as $E_{1}=E_{2}=9.5\;kPa$ . By disrupting the MTs using Nocodazole, the axon elastic modulus was found $E_{i}=1.47\;kPa$ which is taken to be the modulus of the inner region 1. Zhang et al. (2017) performed a coarse-grain molecular dynamic simulation to study the stiffness of the axon membrane and validated the result by AFM experiments (Zhang et al., 2017). They also used an axon of radius 0.5 µm and reported the stiffness of the membrane to be $E_{m}=4.23\;kPa$ . Therefore, we have taken the radius of the axon $R_{a}=0.5\;\mu m$ which gives the volume fraction of the membrane, inner region $\left(R_{a}-R_{c}=200\;nm\right)$ , and cytoskeletal region to be $\alpha_{m}=0.04$ , $\alpha_{i}=0.60$ , and $\alpha_{c}=0.36$ , respectively, for the unmyelinated axon. We can use the inverse rule of mixture often termed as the Reuss model to estimate the transverse elastic modulus of the axon as (Clyne and Hull, 2019)

$$E_{1}={\left[\frac{\alpha_{ms}}{E_{ms}}+\frac{\alpha_{m}}{E_{m}}+\frac{\alpha_{i}}{E_{i}}+\frac{\alpha_{c}}{{C_{11}}_{0}}\right]}^{-1}$$ (32)

For the unmyelinated axon $\left(\alpha_{ms}=0\right)$ and myelinated axon (*g*-ration is varied), the estimated $E_{1}$ is plotted in Figure 15B and compared with the experimental value by varying the $\alpha_{i}$ . For the volume fraction of the inner region 1 of $\alpha_{i}=0.145$ , the estimated transverse modulus equals the experimental value, which is a good estimation. In Equation (32), the elastic modulus of the myelin sheath is defined as $E_{ms}=3K_{ms}\left(1-2\nu_{ms}\right)$ .

### Damage Criteria of Axon Based on the Cytoskeletal Component Failure

**FIGURE 16:**
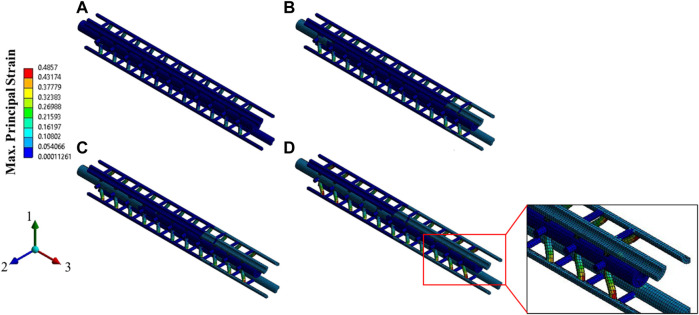
Contour plot of the maximum principal strain (elemental mean) for the strain rate of 5/sec, at **(A)** 2.5% strain, **(B)** 5% strain, **(C)** 7.5% strain, and **(D)** 10% strain (the matrix is hidden to show the strain on the Tau proteins).

In the second part of this manuscript, we have studied the axonal damage threshold based on the cytoskeletal component failure for different loading rates. As mentioned earlier, the load path of a mechanical insult extends from the tissue level to the cellular level and finally to the cytoskeletal level. It is experimentally verified that the maximum local strain in the axon is about only 25% of the tissue level strain (Tamura et al., 2007). This is perhaps due to the undulation of the neuronal axons (Wright, 2012) and the heterogeneity of the cellular microstructure (Montanino and Kleiven, 2018). In this manuscript, we have directly evaluated the cellular level stress and strain by calculating the volume-averaged stress and strain of the RVEs and correlating the cytoskeletal failure as the damage threshold criteria. We have considered the failure strain of the MT and Tau as 50 and 40%, respectively (Janmey et al., 1991)– (Wegmann et al., 2011). A total of 5 loading rates have been introduced in the direction of the MT orientation (direction 3). The strain rates are varied from 10/s to 50/s with a 10/s increment. A total of 25 simulations have been performed for 5 RVE cases (e.g., RVE-1 with $E_{MT}=1.5\;GPa$ , RVE-1 with $E_{MT}=1.9\;GPa$ , RVE-2, RVE-3, and RVE-4). In all simulations, Tau reached its failure strain of 40% much earlier than the MT achieved its 50% failure strain. Montanino et al. (2018) suggested that MT failure strain was never achieved until the axonal strain reached 25% which is significantly higher than the typical axon failure criteria (Montanino and Kleiven, 2018). Similar results were observed by Peter et al. (2012) who predicted cross-link failure than MT failure (Peter and Mofrad, 2012a). As Tau are oriented in the transverse plane (direction 1 and 2) and perpendicular to the loading direction in their reference configuration, we considered the elemental mean of the maximum principal strain of the Tau elements in the FEM model. A representative contour plot of the maximum principal strain of the cytoskeletal components is given in Figure 16. The Tau near the end of the MT showed the maximum strain (see Figure 16D), which is also predicted in (Ahmadzadeh et al., 2014), (Wu et al., 2018), (Wu et al., 2019).

**FIGURE 17:**
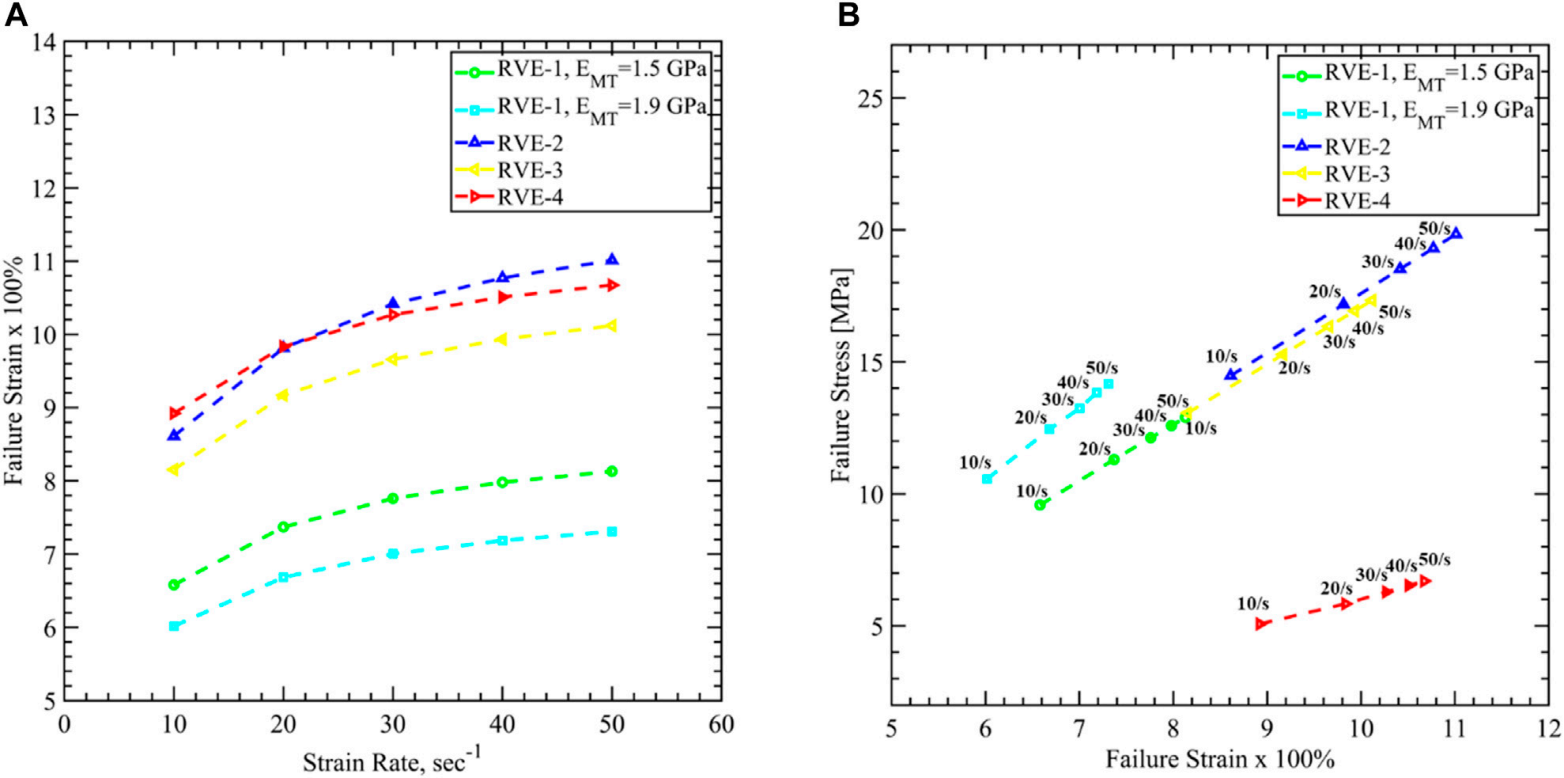
Axon cytoskeletal damage evaluation by mapping damage threshold values for **(A)** axon volume averaged failure strain *vs.* strain rate, and **(B)** axon volume averaged failure stress *vs*. axon volume averaged failure strain for different RVEs. 40% strain is taken as the failure strain of Tau, and corresponding axonal stress and strain are plotted for different loading rates.

In Figure 17A, we have plotted the strain *vs*. strain rate curve for different RVEs. For the Base RVE (i.e., RVE-1 with $E_{MT}=1.5\;GPa$ ) the failure strain varies between 6.6 and 8.1%, which agrees well with the recent studies that identified the neuronal failure due to Tau is 7.3% (Estrada et al., 2021). The axon can fail much earlier as the MT’s stiffness increases to 1.9 GPa. However, decreasing the volume fraction of the MT (i.e., RVE-4) showed the lowest stiffness (see Figure 11F) and hence can stretch up to 8.9–10.6% of strain before the damage initiated *via* Tau failure. The most interesting effect is observed when the volume fraction of Tau is increased in RVE-2 and RVE-3. As we have seen in Figure 11F, increasing $\alpha_{tau}$ increased the short-term stiffness in direction 3. This contradicts the plot in Figure 17A, where we can see higher failure strain in RVE-2 and RVE-3. This is due to the longer relaxation time observed while $\alpha_{tau}$ is increased in Figure 11F. The RVE can stretch more within the time of the loading rates. It is also evident that as RVE-2 showed a longer relaxation time than RVE-3, it can sustain a higher strain before it fails via Tau failure.

Finally, in Figure 17B, we have plotted the failure stress *vs*. failure strain for different RVEs. In experimental observations, both *in-vitro* and *in-vivo*, cellular level stress cannot be identified. The current work can address this issue using the FEM analysis, and the failure stress value can be used for further study in larger FEM models. For the base RVE, the failure stress varies between 9.5 and 12.8 MPa as the strain rate increases. Increasing stiffness of MT shows higher failure stress from 10.5 to 14.1 MPa while decreasing $\alpha_{MT}$ shows lower failure stress varied from 5 to 6.6 MPa. On the other hand, increasing $\alpha_{tau}$ shows higher failure stress from 13 to 19.8 MPa corresponds to higher failure strain, as discussed earlier.

## Conclusion

We have studied the cytoskeletal microstructure of the neuronal axon and developed the RVE to characterize the viscoelastic response. A total of six load cases are needed to estimate nine independent relaxation moduli, and they are summarized in the manuscript for further use in the axon aggregate model. The short time modulus $\left({C_{ij}}_{0}\right)$ is maximum in the longitudinal direction along with the MT orientation ( $C_{33}\left(t\right)$ ). The effect of Young’s modulus of the MT is insignificant in all relaxation moduli except $C_{33}\left(t\right)$ , which is increased as $E_{MT}$ is increased. The softest mode is found when the volume fraction of MT is reduced, corresponding to the shortest relaxation time. Increasing the volume fraction of Tau increases the stiffness and shows a longer relaxation time.

In the second part, we have studied axon damage based on the cytoskeletal component failure. The loading rate is varied between 10/s to 50/s to be consistent with the previous studies. The axon failure strain varies between 6.01 and 11.01%, considering Tau fails at 40% strain. Due to the limitation of achieving conversed FEM solution for the large deformations, all simulations are carried out up to 12% strain of the RVEs for all strain rates. We have not observed the MT reach its failure strain of 50% in this range of stretch. However, Tau failure is a good indication of the structural damage of the axon cytoskeleton, which can lead to functional damage as well. A recent study showed that axon failed at 7.3% strain *via* Tau failure when subjected to the loading rate <100/s, which agrees well with the results shown in this work (Estrada et al., 2021). The failure stress is also estimated and varies between 5 and 19.8 MPa.

As mentioned earlier, previous studies based on neuronal injury had identified both strain and strain rate threshold values; however, Wright (2012) identified that it is difficult to determine the combined effects of strain and strain rate from the available literature (Wright, 2012). Not to mention that it is also challenging to identify the stress level of the damaged axon experimentally *in-vitro* and *in-vivo*. Therefore, this work can address this issue by correlating the axon’s strain to strain rate and the failure stress level at the cellular level, which can be utilized in further studies of axon aggregates using FEM models. However, we need to address that due to the lack of data related to the Tau failure strain as a function of strain rate, we have used a fixed failure strain of 40%. Other than that, the cross-linked network of MT and Tau can also disintegrate by Tau detachment due to phosphorylation. The interfacial strength of Tau binding sites with MT is not included; instead, a perfect bonded condition is used in the FEM study. This also leads to higher stiffness along the MT direction. Other than the structural damage of the cytoskeletal microstructure, there are other forms of event that can lead to diffuse axonal injury (DAI) due to a mechanical insult (Montanino et al., 2021). Morphological damage due to the axonal swelling, mechanoporation through the plasma membrane, and glial cells damage can initiate a complex cascade of molecular events that can further cause cytoskeletal damage, which is also not investigated.

## Data Availability

The raw data supporting the conclusion of this article will be made available by the authors, without undue reservation.
